# NNMT Orchestrates Metabolic‐Epigenetic Reprogramming to Drive Macrophage‐Myofibroblast Transition in Hypertrophic Scarring

**DOI:** 10.1002/advs.202502727

**Published:** 2025-11-10

**Authors:** Xiwen Dong, Weinan Guo, Yuxuan Qian, Wenyan Jin, Xiaozhen Li, Jingyuan Yang, Qingrong Ni, Shuang Wu, Fanni Li, Hua Wang, Chunying Li, Hong Cai

**Affiliations:** ^1^ Department of Dermatology Air Force Medical Center Beijing 100039 P. R. China; ^2^ Department of Dermatology Xijing Hospital Fourth Military Medical University Xi'an Shaanxi 710032 P. R. China; ^3^ Department of High Talent The First Affiliated Hospital of Xi'an Jiaotong University Xi'an Shaanxi 710061 P. R. China; ^4^ Beijing Institute of Radiation Medicine Beijing 100142 P. R. China

**Keywords:** hypertrophic scar (HS), macrophage‐myofibroblast transition (MMT), nicotinamide N‐methyltransferase (NNMT), paired related homeobox (PRRX1)

## Abstract

Hypertrophic scar (HS) is a cutaneous fibrotic disorder characterized by persistent myofibroblast activation and excessive extracellular matrix deposition. Elucidating the origin and characteristics of myofibroblasts remains a central focus in the field. This study identifies a novel subtype of scar myofibroblasts originating from macrophage‐myofibroblast transition (MMT). MMT cells constitute a significant proportion of HS myofibroblasts and drive HS progression. Multi‐omics analysis uncovered nicotinamide N‐methyltransferase (NNMT) as a metabolic orchestrator of MMT. Liquid chromatograph mass spectrometer reveals NNMT‐mediated depletion of nicotinamide adenine dinucleotide (NAD^+^) and *S*‐adenosyl methionine(SAM), triggering H3K27ac accumulation and H3K27me3 loss. This epigenetic reprogramming facilitated the expression of master transcription factor paired‐related homeobox 1 (Prrx1) and its nuclear co‐condensation with super‐enhancer (SE)  components. Inhibition of NNMT disrupted Prrx1‐SE interactions, suppressed MMT in vitro, and reduced scar volume in vivo. This study 1) identifies a new origin of scar‐associated myofibroblasts, 2) establishes metabolite‐guided epigenetic alteration as a regulator of myofibroblasts cellular plasticity, and 3) nominates NNMT as a therapeutic target for HS and related fibrotic disorders.

## Graphical abstract

In macrophage‐myofibroblast transition, upregulated NNMT depletes *S*‐Adenosylmethionine‌ (SAM) and nicotinamide adenine dinucleotide(NAD^+^), thereby triggering epigenetic reprogramming via Histone H3 Lysine 27 acetylation (H3K27ac) accumulation at the promoter region of master transcription factor Prrx1. This chromatin remodeling drives Prrx1 overexpression, enhancing its binding to super‐enhancers to activate a pro‐fibrotic transcriptional program that promotes myofibroblast lineage commitment and hypertrophic scar progression.

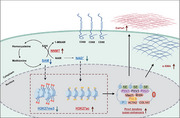

## 1 Introduction

Hypertrophic scars (HS) are common skin fibrotic conditions caused by abnormal wound healing processes after physical or chemical injuries.^[^ ^1^ ^]^ HS can cause disfigurement, restricted movement, and permanent functional impairment, leading to psychological disorders such as anxiety and depression.^[^ ^2^ ^]^ Considering the sequelae caused by HS physically and mentally, HS has been identified as a significant unmet challenge after cutaneous trauma.^[^ ^3^ ^]^ Approaches to treat HS include corticosteroid injection, laser treatment, radio therapy and surgical revision. However, an ideal therapeutic approach that effectively minimizes scar formation and recurrence has yet to be developed. Therefore, a comprehensive understanding of the molecular mechanisms governing scar formation is crucial for advancing targeted therapeutic strategies.

The pathogenesis of HS is fundamentally rooted in dysregulated wound healing processes, characterized by persistent myofibroblast activation and excessive deposition of collagen fibers and extracellular matrix (ECM) components.^[^ ^4^ ^]^ Elucidating the cellular origins and molecular mechanisms underlying these dysregulated myofibroblasts has remained a central focus and critical challenge in HS research. Through comprehensive analysis of single‐cell RNA sequencing (scRNA‐seq) data and HS tissue specimens, we have identified macrophage‐myofibroblast transition (MMT) as a significant source of scar‐associated myofibroblasts. MMT cells are characterized by the dual expression of canonical macrophage marker cluster of differentiation (CD68) and myofibroblast‐specific marker α‐smooth muscle actin (α‐SMA).^[^ ^5^, ^6^, ^7^, ^8^ ^]^ Notably, MMT‐derived myofibroblasts constitute a substantial proportion of the myofibroblast population in HS lesions and actively contribute to scar progression. However, the precise regulatory mechanisms governing MMT remain to be fully elucidated.

Accumulating evidence from previous studies has highlighted the pivotal role of metabolic reprogramming and epigenetic modifications in regulating myofibroblast activation and persistence.^[^ ^9^ ^]^ Metabolic networks orchestrate the intricate processing of nutrients to meet the dynamic energy demands and biosynthetic requirements of various cellular processes.^[^ ^10^ ^]^ Notably, metabolic intermediates function as essential substrates and cofactors for chromatin‐modifying enzymes, thereby establishing a direct molecular link between cellular metabolic status and epigenetic regulation.^[^ ^11^ ^]^ Our study identified nicotinamide N‐methyltransferase (NNMT) as a critical regulator of MMT. NNMT catalyzes the methylation of nicotinamide (NAM) using *S*‐adenosylmethionine (SAM) as the methyl donor, generating 1‐methylnicotinamide (1‐MNAM) and *S*‐adenosyl‐L‐homocysteine (SAH).^[^ ^12^ ^]^ This reaction simultaneously reduces cellular levels of both SAM and nicotinamide adenine dinucleotide (NAD^+^), establishing a dual metabolic constraint that influences multiple cellular processes such as epigenetic modifications.^[^ ^13^ ^]^

In this study, we employed an integrative approach combining scRNA‐seq analysis and HS tissue examination to elucidate the profibrotic role of MMT in scar pathogenesis. Through gene expression intervention, we identified NNMT a central regulator of MMT and delineated its dual metabolic and epigenetic regulatory mechanisms. Furthermore, we discovered that the master transcriptional factor (mTF) paired related homeobox 1 (Prrx1) functions as a key downstream effector of NNMT signaling in MMT. Most significantly, inhibition of NNMT in MMT cells effectively attenuated scar progression, establishing NNMT as a promising therapeutic target for anti‐fibrotic intervention.

## 2 Results

### 2.1 MMT Cells Contribute to the Myofibroblast Population in HS

**Figure 1:**
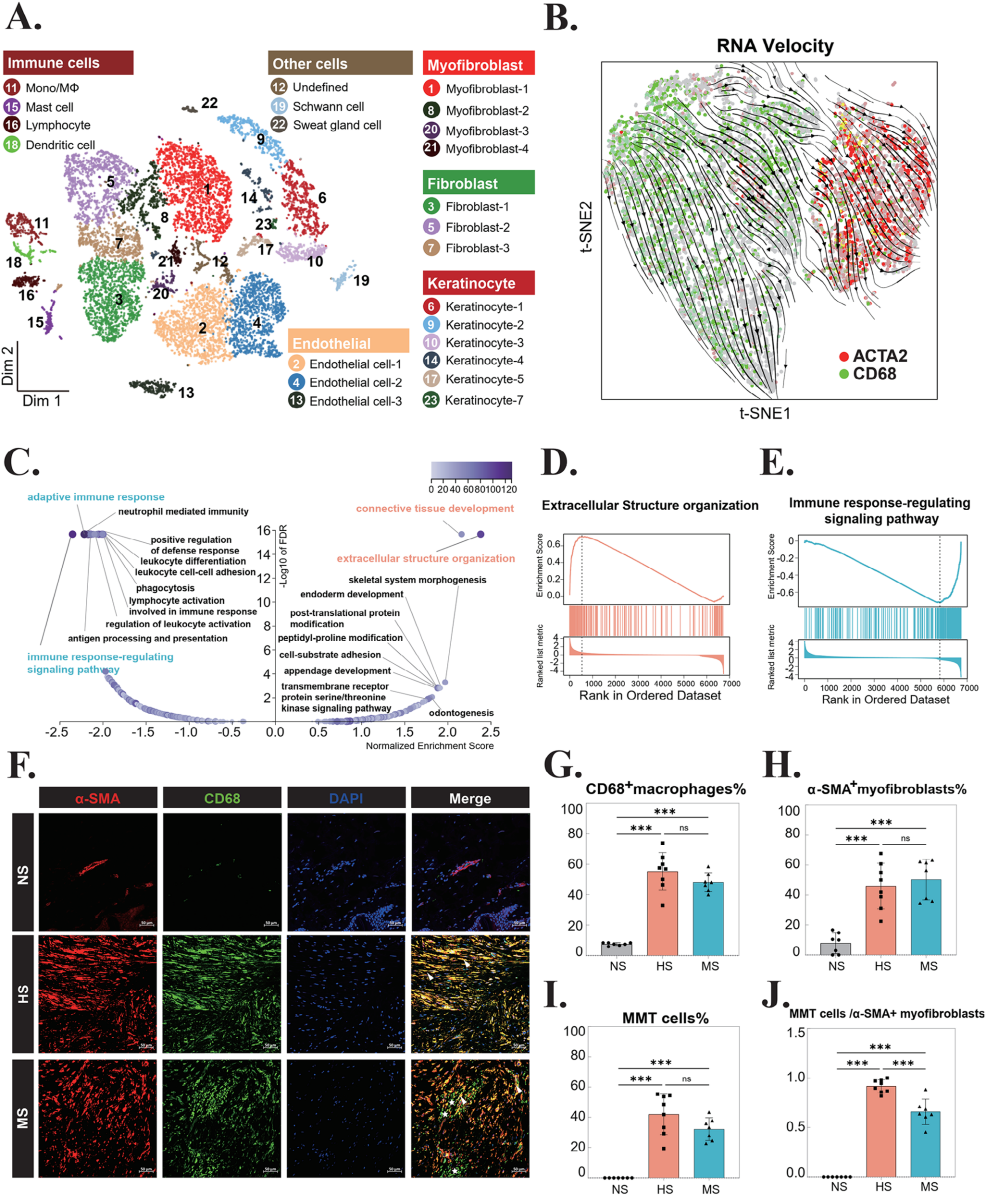
Identification of Macrophage‐Myofibroblast Transition (MMT) Cells in Hypertrophic Scar (HS). A) t‐distributed Stochastic Neighbor Embedding (t‐SNE) visualization of single‐cell RNA sequencing (scRNA‐seq) data. Twenty‐three distinct cellular clusters are color‐coded, with the general identity of each cluster annotated on the graph. B) RNA velocity vectors overlaid on the t‐SNE map, illustrating the trajectory of cells expressing *CD68* (green) and *ACTA2* (red). C) Volcano plot showing the results of Gene Set Enrichment Analysis (GSEA) using the GO‐Biological Process database. D) Enrichment plot for the GO term GO:0043062, highlighting genes involved in extracellular structure organization. E) Enrichment plot for the GO term GO:0043062, highlighting genes associated with immune response‐regulating signaling pathways. F) Immunofluorescence staining of α‐SMA (red) and CD68 (green) in normal skin (NS), HS, and mature scar (MS) tissues. MMT cells are indicated by co‐localization of α‐SMA and CD68 (arrows). Pentagrams denote CD68 single‐positive macrophages. Scale bar = 50 µm. n = 7. G) Quantification of CD68^+^ macrophages in NS, HS, and MS samples. ***p < 0.001; ns, not significant. n = 7. H) Quantification of α‐SMA^+^ myofibroblasts in NS, HS, and MS samples. ***p < 0.001; ns, not significant. n = 7. I) Quantification of CD68^+^ and α‐SMA^+^ MMT cells in NS, HS, and MS samples. ***p < 0.001; ns, not significant. n = 7. J) Bar graph showing the ratio of MMT cells to total myofibroblasts in NS, HS, and MS samples. ***p < 0.001. n = 7. Abbreviations: Mono, monocyte; MΦ, macrophage; t‐SNE, t‐distributed Stochastic Neighbor Embedding; NS, normal skin; HS, hypertrophic scar; MS, mature scar; MMT, macrophage‐myofibroblast transition; *ACTA2*, actin alpha 2, smooth muscle, aorta; *CD68*, cluster of differentiation 68.

To investigate the presence of MMT cells in HS, we performed comprehensive analysis of scRNA‐seq data. Following rigorous quality control, normalization, and batch effect correction (Table S1; Figure S1, Supporting Information), we identified 23 distinct cell clusters through t‐distributed Stochastic Neighbor Embedding (t‐SNE) visualization (**Figure** **1**A). Cluster‐specific marker genes were systematically characterized and presented in Figures S2 and S3 (Supporting Information). Based on established criteria from previous studies,^[^ ^7^ ^]^ we identified MMT cells via co‐expression analysis of canonical markers, including *ACTA2* (encoding α‐smooth muscle actin, α‐SMA) for myofibroblasts and CD68 for macrophages (Figure S4A–C, Supporting Information). MMT cells were predominantly located within myofibroblast‐enriched clusters (cluster 1, 8, and 21) and fibroblast populations (cluster 5 and 7), accounting for 34.7% of *ACTA2*‐positive cells. Notably, spatial distribution features of MMT cells were obviously different from fibrocytes within the cellular landscape of HS in the t‐SNE map (Figure S4D–F, Supporting Information).

To delineate the cellular transition dynamics among macrophages, MMT cells, and myofibroblasts, we performed RNA velocity analysis. By integrating RNA velocity vectors with *CD68* and *ACTA2* expression patterns in t‐SNE space, we identified distinct differentiation trajectories originating from macrophages (*CD68* ^+^) and progressing to either MMT cells (*CD68* ^+^ and *ACTA2* ^+^) or myofibroblasts (*ACTA2* ^+^) (Figure 1B; Figure S4G,H, Supporting Information).

**Figure 2:**
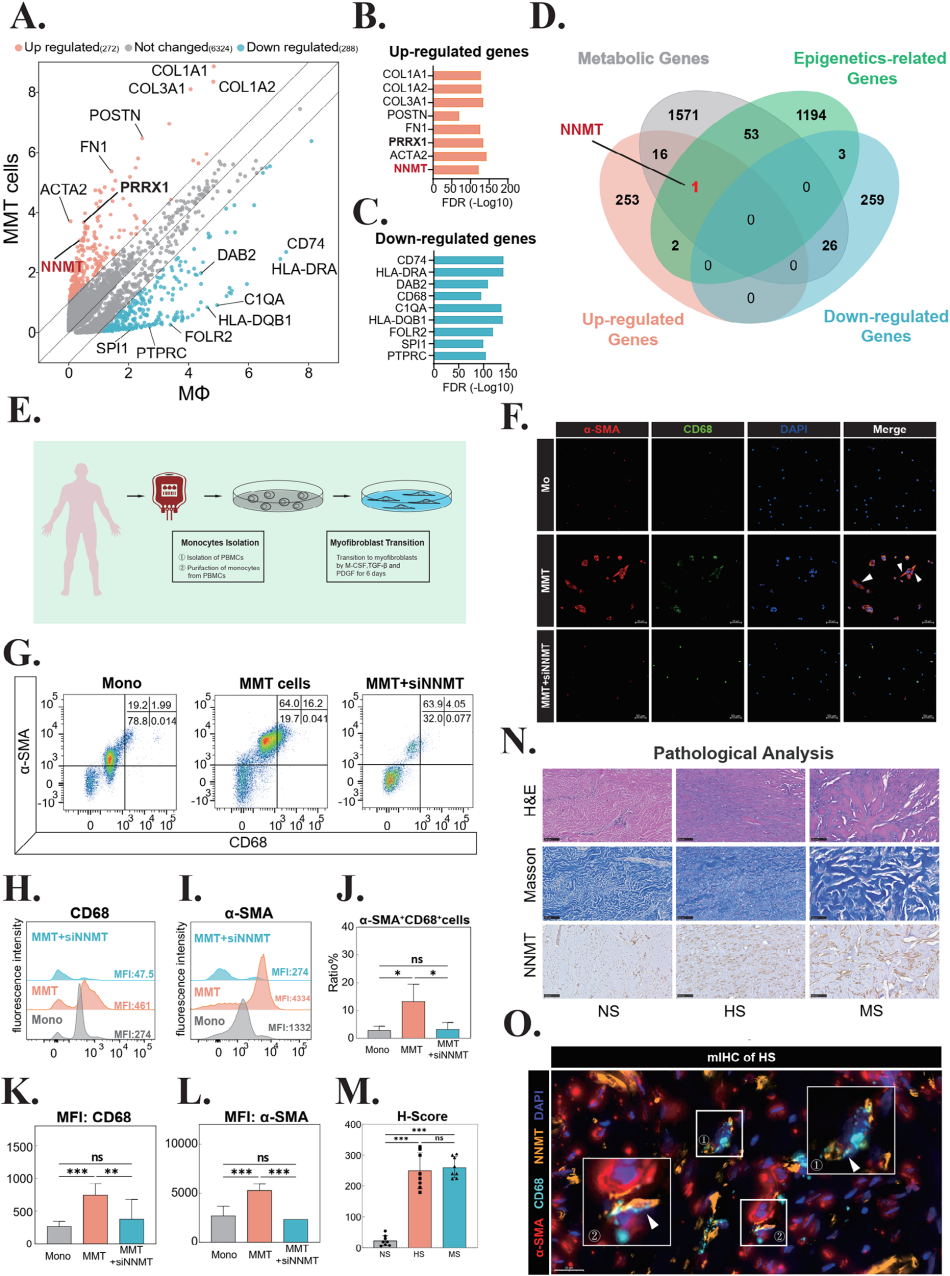
NNMT Drives Macrophage‐Myofibroblast Transition (MMT) Through Metabolic and Epigenetic Regulation. A) Scatter plot comparing gene expression fold changes between MMT cells and macrophages, highlighting *NNMT* (red) as a key differentially expressed gene. B) False discovery rate (FDR) analysis of upregulated genes in MMT cells. C) FDR analysis of downregulated genes in MMT cells. D) Venn diagram illustrating the overlap between upregulated/downregulated genes in MMT cells, KEGG metabolic genes, and KEGG epigenetic‐related genes, with *NNMT* identified as the intersection candidate. E) Schematic of the in vitro induction protocol for differentiating monocytes into MMT cells. F) Immunofluorescence images of induced MMT cells and MMT + siNNMT, showing co‐expression of α‐SMA (red) and CD68 (green). White arrows indicate MMT cells; scale bar = 50 µm. n = 3. G) Flow cytometry analysis of monocytes, MMT cells, and MMT + siNNMT (NNMT knockdown). Quadrant plots display CD68 (x‐axis) versus α‐SMA (y‐axis), with quadrant percentages shown in the top‐right corner. n = 3. H) Quantification of CD68 fluorescence intensity across experimental groups. n = 3. I) Quantification of α‐SMA fluorescence intensity across experimental groups. n = 3. J) Proportion of CD68^+^ and α‐SMA^+^ double‐positive cells. Groups: Mono (monocytes), MMT (MMT cells), MMT + siNNMT (NNMT‐interfered MMT cells). n = 3. K) Statistical analysis of CD68 mean fluorescence intensity (MFI). **p < 0.01; ***p < 0.001; ns, not significant. n = 3. L) Statistical analysis of α‐SMA MFI. ***p < 0.001; ns, not significant. n = 3. M) Histological score (H‐score) of NNMT expression in normal skin (NS), hypertrophic scar (HS), and mature scar (MS). ***p < 0.001; ns, not significant. n = 7. N) Representative images of H&E, Masson's trichrome, and NNMT immunohistochemistry (IHC) staining in NS, HS, and MS tissues. Scale bar = 100 µm. n = 7. O) Multiplex IHC staining showing co‐localization of α‐SMA (red), CD68 (cyan), and NNMT (orange) in MMT cells (white arrows). Scale bar = 20 µm. n = 3. Abbreviations: MMT, macrophage‐myofibroblast transition; *COL1A1*, collagen type I alpha 1 chain; *COL3A1*, collagen type III alpha 1 chain; *COL1A2*, collagen type I alpha 2 chain; *POSTN*, periostin; *FN1*, fibronectin 1; *ACTA2*, actin alpha 2, smooth muscle, aorta; *NNMT*, Nicotinamide N‐Methyltransferase; *PRRX1*, paired related homeobox 1; *DAB2*, disabled 2; *CD74*, cluster of differentiation 74; *HLA‐DRA*, major histocompatibility complex, class II, DR alpha; *C1QA*, complement C1q subcomponent a; *HLA‐DQB1*, major histocompatibility complex, class II, DQ beta 1; *FOLR2*, folate receptor 2; *SP1*, Sp1 Transcription Factor; *PTPRC*, protein tyrosine phosphatase receptor type C; FDR, false discovery rate; Mono, monocyte; M‐CSF, macrophage colony‐stimulating factor; TGF‐β, transforming growth factor beta; PDGF, platelet‐derived growth factor; α‐SMA, alpha‐smooth muscle actin; DAPI, 4′,6‐Diamidino‐2‐Phenylindole; NS, normal skin; HS, hypertrophic scar; MS, mature scar; H&E, Hematoxylin and Eosin staining; MFI, median fluorescence intensity.

Gene set enrichment analysis (GSEA) revealed significant pathway alterations during this transition. Compared to macrophages, MMT cells exhibited upregulation of genes associated with “extracellular structure organization” and “connective tissue development” (Figure 1C,D), consistent with their profibrotic phenotype. Conversely, genes involved in “immune response‐regulating signaling pathway” and “adaptive immune response” were significantly downregulated (Figure 1C–E), indicating a recession of immune regulatory functions. These findings were further validated using the Reactome database, which showed consistent pathway enrichment patterns (Figure S4I–K, Supporting Information).

To validate our scRNA‐seq findings, we performed immunofluorescence (IF) staining and confocal microscopy analysis of normal skin (NS), HS and mature scar (MS) tissue specimens. In HS tissues, MMT cells exhibited characteristic spindle‐shaped morphology typical of activated myofibroblasts (Figure 1F, arrows), a phenotype rarely observed in NS samples (Figure 1F,I). Quantitative analysis revealed that MMT cells accounted for 42.0% of total cells in HS tissues (Figure 1I). In contrast, MS tissues showed significantly reduced MMT cell proportions, comprising only 32.2% of total cells and 65.8% of α‐SMA^+^ myofibroblasts (Figure 1J). Notably, MS tissues displayed scattered CD68^+^ macrophages (Figure 1F, pentagrams), suggesting a potential shift in cellular composition during scar maturation. These findings strongly support the hypothesis that MMT cells play a significant role in scar fibrogenesis and tissue remodeling.

### 2.2 NNMT Serves as a Key Metabolic and Epigenetic Regulator of MMT

Emerging evidence has highlighted the critical role of metabolic reprogramming and epigenetic regulation in myofibroblast activation.^[^ ^9^ ^]^ To identify potential regulators of MMT, we performed differential gene expression analysis comparing MMT cells with their macrophage precursors. This analysis revealed 272 significantly upregulated genes and 288 downregulated genes in MMT cells (**Figure** **2**A–C). Through integrative analysis of epigenetic‐related genes (KEGG pathway hsa03036), metabolic genes (KEGG database), and MMT‐upregulated genes, we identified *NNMT* as a potential key regulator of MMT (Figure 2D). Spatial expression analysis using t‐SNE visualization demonstrated that *NNMT* was predominantly localized within fibroblast and myofibroblast clusters, with significantly elevated expression levels in MMT cells compared to other cell populations (Figure S5A,B, Supporting Information).

**Figure 3:**
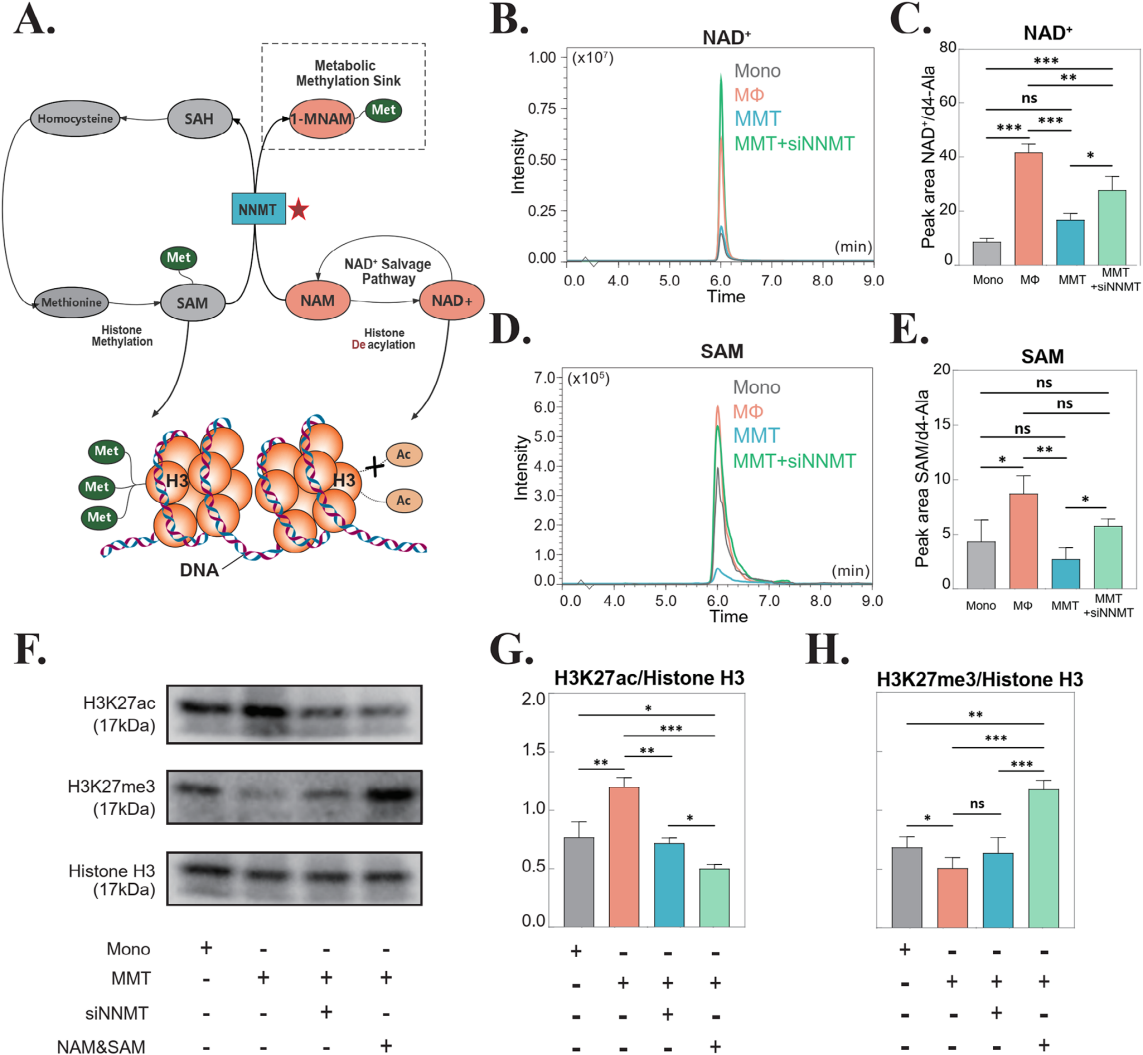
NNMT Modulates Metabolic and Epigenetic Pathways in MMT Cells. A) Schematic diagram illustrating the biological role of nicotinamide N‐methyltransferase (NNMT) in regulating metabolite levels and histone modifications. B) Representative extracted‐ion chromatogram (EIC) of nicotinamide adenine dinucleotide (NAD^+^) in MMT cells. n = 3. C) Quantification of NAD^+^ levels normalized to the internal standard d4‐Ala. *p < 0.05; **p < 0.01; ***p < 0.001; ns, not significant. n = 3. D) Representative EIC of *S*‐adenosylmethionine (SAM). n = 3. E) Quantification of SAM levels normalized to d4‐Ala. *p < 0.05; **p < 0.01; ***p < 0.001; ns, not significant. n = 3. F) Western blot analysis of H3K27ac, H3K27me3, and histone H3 expression in MMT cells under indicated treatments. For metabolite supplementation, cells were cultured with 1 mmol L^−1^ nicotinamide (NAM) and 0.1 mmol L^−1^ SAM. n = 3. G) Quantification of H3K27ac levels relative to histone H3. *p < 0.05; **p < 0.01; ***p < 0.001; ns, not significant. n = 3. H) Quantification of H3K27me3 levels relative to histone H3. *p < 0.05; **p < 0.01; ***p < 0.001; ns, not significant. n = 3. Abbreviations: NNMT, nicotinamide N‐methyltransferase; siNNMT, small interfering RNA targeting NNMT; HS, hypertrophic scar; MS, mature scar; SAM, *S*‐adenosylmethionine; SAH, *S*‐adenosylhomocysteine; NAM, nicotinamide; 1‐MNAM, 1‐methylnicotinamide; NAD^+^, nicotinamide adenine dinucleotide; Met, methyl group; Ac, acetyl group; H3, histone H3; DNA, deoxyribonucleic acid; Mono, monocyte; MΦ, macrophage; MMT, macrophage‐myofibroblast transition; d4‐Ala, 4′‐aminolevulinic acid‐d4; H3K27ac, histone H3 lysine 27 acetylation; H3K27me3, histone H3 lysine 27 trimethylation.

To further investigate the functional role of NNMT in MMT, we established in vivo models using both Peripheral Blood Mononuclear Cell (PBMC)‐derived monocytes and THP‐1 cells, followed by targeted *NNMT* gene interference (Figure 2E; Figure S5C, Supporting Information). Following MMT induction, we observed the emergence of characteristic spindle‐shaped myofibroblasts (Figure S5D,E, Supporting Information, black arrows), which were subsequently confirmed as bona fide MMT cells through dual‐positive staining for α‐SMA and CD68 using both immunocytochemistry (ICC) staining and flow cytometry (Figure 2F‐L; Figure S5F, Supporting Information), consistent with established MMT criteria.^[^ ^5^ ^]^ Notably, NNMT inhibition resulted in significant suppression of MMT, as evidenced by three distinct phenotypic alterations: 1) loss of characteristic spindle‐shaped morphology (Figure 2F; Figure S5D–F, Supporting Information), 2) substantial reduction in MMT cell numbers (Figure 2J), and 3) marked downregulation of α‐SMA expression (Figure 2I,L). These findings collectively demonstrate the critical role of NNMT in mediating MMT.

Furthermore, immunohistochemical (IHC) analysis of scar tissue specimens revealed robust cytoplasmic expression of NNMT in scar‐associated myofibroblasts (Figures 2M,N). In HS, NNMT‐positive myofibroblasts were predominantly distributed throughout the fibrotic regions. In contrast, MS exhibited a distinct spatial organization, with NNMT‐expressing myofibroblasts aligning parallel to the dense collagen fiber bundles (Figure 2N). Multiplex IHC (mIHC) analysis further confirmed significant upregulation of NNMT expression specifically in MMT cells within HS tissue (Figure 2O). These findings collectively establish NNMT as a critical metabolic and epigenetic regulator driving MMT progression in pathological scarring.

### 2.3 NNMT‐Mediated Metabolic and Epigenetic Regulation During MMT

NNMT exerts its biological function through catalyzing the methylation of NAM, thereby reducing two crucial metabolic substrates: SAM, the universal methyl donor for methylation reactions, and NAD^+^, an essential cofactor for deacetylation reactions (**Figure** **3**A). To investigate the metabolic consequences of NNMT activity during MMT, we performed targeted liquid chromatography‐mass spectrometry (LC‐MS) analysis. Representative extracted ion chromatograms (EICs) revealed a significant reduction in intracellular NAD^+^ levels following MMT induction compared to macrophage controls (Figure 3B,C). Notably, NNMT knockdown (siNNMT) effectively rescued this NAD^+^ depletion (Figure 3B,C). Similarly, we observed a marked decrease in SAM levels during MMT progression, which was also reversed by NNMT inhibition (Figure 3D,E).

As a key epigenetic regulatory mechanism, histone modification is highly sensitive to fluctuations in cellular metabolite concentrations.^[^ ^11^ ^]^ Our investigation revealed significant alterations in histone H3K27 modifications during MMT progression. Specifically, we observed increased acetylation at H3K27 (H3K27ac) during MMT (Figure 3F,G), which was effectively attenuated by either NNMT inhibition or nicotinamide (NAM) supplementation (Figure 3F,G). Conversely, trimethylation of H3K27 (H3K27me3) showed a marked decrease during MMT, a phenomenon that could be reversed by SAM supplementation (Figure 3F,H). Interestingly, NNMT inhibition resulted in a non‐significant increase in H3K27me3 levels (Figure 3H). This observation suggests a complex regulatory relationship.

In addition, we examined the impact of NNMT perturbation on other histone modifications associated with transcriptional activation, including H3K4me3 (a marker of active promoters) and H3K9ac (linked to open chromatin). Notably, NNMT interference led to a significant reduction in H3K9ac levels, whereas H3K4me3 remained stable (Figure S6A, Supporting Information), suggesting selective epigenetic regulation by NNMT.

Further analysis revealed a concomitant decrease (after MMT induction) in Sirtuin1 (Sirt1), a NAD^+^‐dependent histone deacetylase, likely explaining the reduction in H3K9ac and H3K27ac levels, as Sirt1 directly modulates these modifications(Figure S6B, Supporting Information). In contrast, the expression of enhancer of zeste homolog 2‌ (EZH2), the catalytic subunit of PRC2 responsible for H3K27me3 deposition, was unaltered, indicating that NNMT's effects may be mediated through EZH2 activity alteration rather than transcriptional regulation (Figure S6B, Supporting Information).^[^ ^14^ ^]^ Collectively, these findings demonstrate that NNMT drives MMT by metabolically regulating NAD^+^ and SAM pools, and preferentially modulating H3K27.

### 2.4 NNMT Regulates the Expression of mTF Prrx1

**Figure 4:**
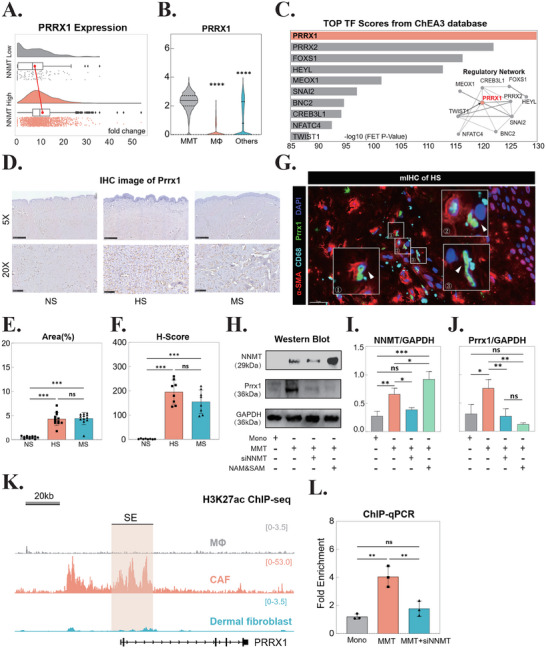
NNMT Orchestrates a Metabolo‐Epigenetic Axis to Drive PRRX1 Expression in MMT. A) Raincloud plot comparing *PRRX1* expression between *NNMT*‐high and *NNMT*‐low groups in MMT cells. B) Violin plot showing *PRRX1* expression across cell populations: MMT cells, macrophages (MΦ), and other cell types. C) Top 10 transcription factors (TFs) predicted to regulate MMT‐associated genes by ChEA3 TF‐target analysis. Bar charts show TF enrichment scores, with insets depicting local TF‐gene interaction networks. D) Immunohistochemistry (IHC) staining of Prrx1 in normal skin (NS), hypertrophic scar (HS), and mature scar (MS). Scale bars = 500 µm (5×) and 100 µm (20×). n = 7. E) Quantification of Prrx1‐positive area in IHC‐stained tissues. ***p < 0.001; ns, not significant. n = 7. F) Histological score (H‐score) of Prrx1 expression in NS, HS, and MS. ***p < 0.001; ns, not significant. n = 7. G) Multiplex IHC staining showing co‐localization of α‐SMA (red), CD68 (cyan), and Prrx1 (green) in MMT cells (white arrows). Scale bar = 20 µm. n = 3. H) Western blot analysis of NNMT, Prrx1, and GAPDH in MMT cells treated with NAM (1 mmol L^−1^) and SAM (0.1 mmol L^−1^). n = 3. I) Quantification of NNMT protein levels normalized to GAPDH. *p < 0.05; **p < 0.01; ns, not significant. n = 3. J) Quantification of Prrx1 protein levels normalized to GAPDH. *p < 0.05; **p < 0.01; ns, not significant. n = 3. K) H3K27ac ChIP‐seq tracks at the PRRX1 locus in macrophages, cancer‐associated fibroblasts (CAFs), and dermal fibroblasts. Super‐enhancers (SEs) are highlighted. Y‐axis scales: 0–3.5 (macrophages), 0–53.0 (CAFs), 0–3.5 (fibroblasts). L) ChIP‐qPCR validation of H3K27ac enrichment at the Prrx1 SE region. **p < 0.01; ns, not significant. n = 3. Abbreviations: *NNMT*, nicotinamide N‐methyltransferase; MMT, macrophage‐myofibroblast transition; MΦ, macrophage; PRRX1, paired related homeobox 1; *FOXS1*, forkhead box S1; *HEY*, hairy/enhancer‐of‐split related with YRPW motif; *LMOX1*, LIM homeobox 1; *SNAI2*, snail family transcriptional repressor 2; *BNC2*, basonuclin 2; *CREB3L1*, cAMP responsive element binding protein 3 like 1; *NFATC4*, nuclear factor of activated T‐cells, cytoplasmic 4; *TWIST1*, twist family BHLH transcription factor 1; IHC, immunohistochemistry; NS, normal skin; HS, hypertrophic scar; MS, mature scar; CD68, cluster of differentiation 68; α‐SMA, alpha‐smooth muscle actin; DAPI, 4′,6‐diamidino‐2‐phenylindole; GAPDH, glyceraldehyde‐3‐phosphate dehydrogenase; H3K27ac, histone H3 lysine 27 acetylation; kDa, kilodalton; ChIP‐seq, chromatin immunoprecipitation sequencing; ChIP‐qPCR, chromatin immunoprecipitation quantitative PCR; CAF, cancer‐associated fibroblast.

Transcriptomic analysis of scRNA‐seq data revealed *PRRX1* was significantly upregulated in NNMT‐high cell populations (Figures 2A and **4**A). Spatial expression mapping using t‐SNE visualization demonstrated predominant *PRRX1* localization within fibroblast and myofibroblast clusters, with particularly elevated expression in MMT cells (Figure 4B; Figure S6C, Supporting Information). Notably, computational network analysis identified PRRX1 as a central regulator in the transcription factor network governing MMT‐specific gene expression patterns (Figure 4C).

IHC analysis confirmed increased Prrx1 protein expression in HS tissues, showing predominant nuclear localization in myofibroblasts, consistent with its role as a transcription factor (Figure 4D–F). In contrast, MS tissues exhibited dual cytoplasmic and nuclear Prrx1 localization patterns (Figure 4D–F). mIHC further validated the nuclear enrichment of Prrx1 specifically in MMT cells within HS tissues (Figure 4G), supporting its functional role in MMT regulation.

In vivo MMT models demonstrated significant upregulation of Prrx1 expression, which was effectively attenuated by either NNMT inhibition or NAM and SAM supplementation, suggesting that Prrx1 expression is regulated by NNMT‐mediated metabolic alterations (Figure 4H,I). To investigate the underlying epigenetic mechanisms, we designed quantitative PCR (qPCR) primers targeting the promoter region of PRRX1, which encompasses a super‐enhancer (SE) sequence (Figure 4K; Tables S1 and S2, Supporting Information). Chromatin immunoprecipitation followed by qPCR (ChIP‐qPCR) analysis revealed significant enrichment of H3K27ac at the PRRX1 promoter region during MMT progression, with markedly reduction following NNMT inhibition (Figure 4L). These findings establish a mechanistic link between NNMT activity and Prrx1 expression, demonstrating that NNMT promotes PRRX1 transcription through H3K27ac‐mediated epigenetic activation of its promoter region.

### 2.5 Prrx1‐SE Binding Drives MMT

**Figure 5:**
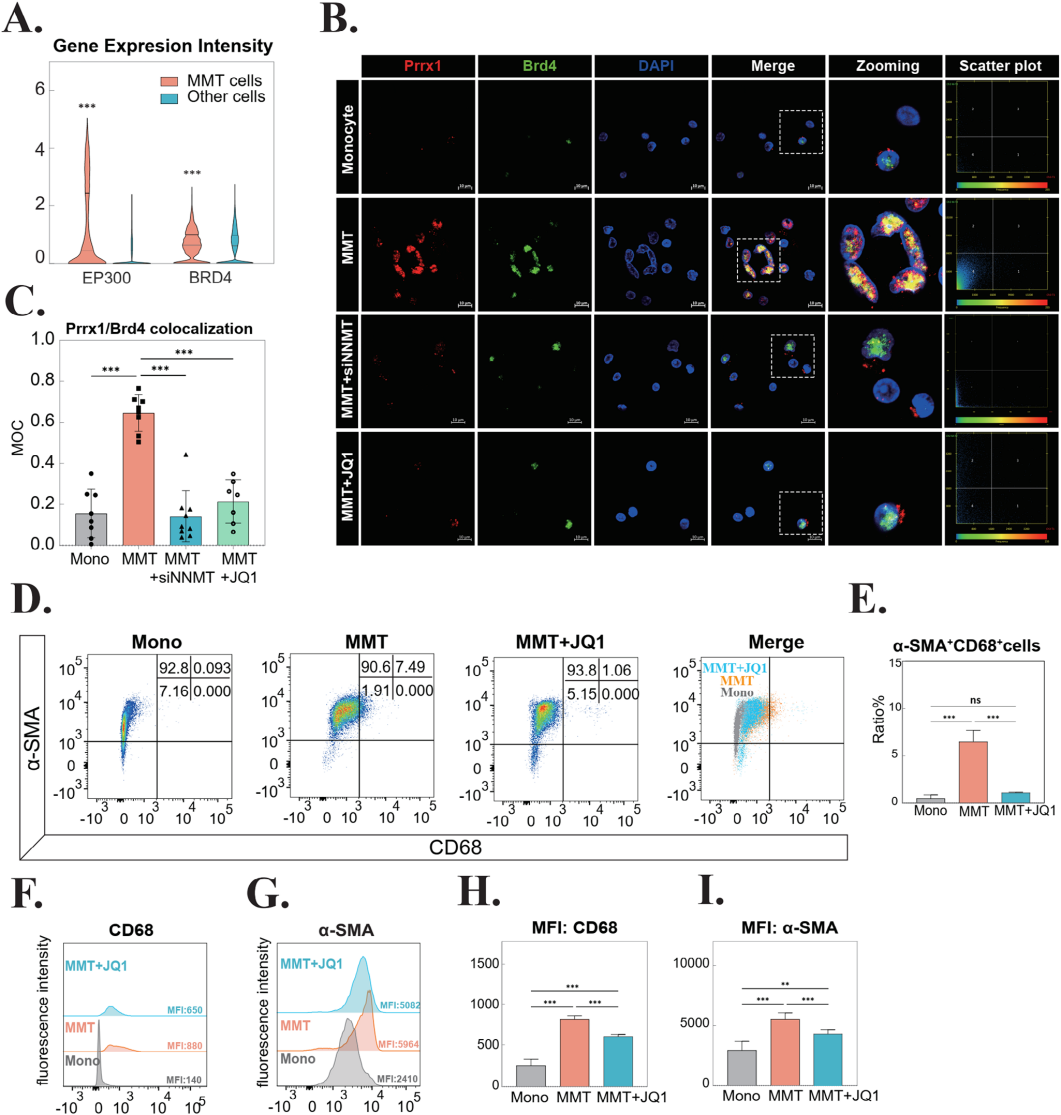
PRRX1 Binds Super‐Enhancers (SEs) to Drive Macrophage‐Myofibroblast Transition (MMT). A) mRNA expression levels of EP300 and BRD4 (key SE‐associated factors) in MMT cells compared to other cell populations. B) Immunofluorescence imaging showing nuclear co‐localization of PRRX1 (red) and BRD4 (green). White boxes indicate enlarged regions, with scatter plots depicting intensity correlations (x‐axis: PRRX1; y‐axis: BRD4). Cells were treated with 500 nmol L^−1^ JQ1 (BET inhibitor) during MMT induction. n = 3. C) Quantification of nuclear co‐localization using Mander's overlap coefficient (MOC). ***p < 0.001. n = 3. D) Flow cytometry analysis of CD68 (x‐axis) and α‐SMA (y‐axis) in monocytes (grey), MMT cells (orange), and MMT + JQ1 cells (blue). Quadrant percentages are shown in the top‐right corner. n = 3. E) Proportion of CD68 and α‐SMA double‐positive MMT cells across experimental groups. ***p < 0.001; ns, not significant. n = 3. F) Fluorescence intensity of CD68 in flow cytometry analysis. n = 3. G) Fluorescence intensity of α‐SMA in flow cytometry analysis. n = 3. H) Quantification of CD68 median fluorescence intensity (MFI). ***p < 0.001; ns, not significant. n = 3. I) Quantification of α‐SMA MFI. ***p < 0.001; ns, not significant. n = 3. Abbreviations: Mono, monocyte; MMT, macrophage‐myofibroblast transition; PRRX1, paired related homeobox 1; α‐SMA, alpha‐smooth muscle actin; CD68, cluster of differentiation 68; EP300, E1A binding protein P300; BRD4, bromodomain‐containing protein 4; MFI, median fluorescence intensity; SE, super‐enhancer; BET, bromodomain and extra terminal.

Previous studies have established Prrx1 as a mTF that orchestrates myofibroblast lineage transition through binding to SEs.^[^ ^15^ ^]^ To validate this mechanism in MMT, we first analyzed the expression profile of SE‐associated cofactors. ScRNA‐seq analysis revealed significant upregulation of key SE components, including EP300, BRD4, CREBBP, and MED1, in MMT cells, as evidenced by both increased expression ratios and intensity levels (**Figure** **5**A; Figure S6D,E, Supporting Information).

Furthermore, we investigated the formation of transcriptional condensates through phase separation, a hallmark of SE activity.^[^ ^15^, ^16^ ^]^ Confocal microscopy demonstrated nuclear co‐localization of Prrx1 and Brd4 in MMT cells, with a Mander's overlap coefficient (MOC) of 0.65, as confirmed by intensity scatter plot analysis (Figure 5B,C). Notably, both NNMT inhibition and SE disruption using the BET inhibitor JQ1 significantly reduced Prrx1‐Brd4 co‐localization (Figure 5B,C; Figure S5G, Supporting Information), underscoring the critical role of NNMT in facilitating Prrx1‐SE interactions and transcriptional complex formation.

Significantly, pharmacological disruption of Prrx1‐SE interactions by JQ1 or interfering Prrx1 expression resulted in a dramatic reduction in MMT induction efficiency, accompanied by substantial downregulation of α‐SMA expression (Figure 5D‐I; Figure S5F, Supporting Information). These findings establish a direct mechanistic link between Prrx1‐SE interaction and MMT progression, while demonstrating the regulatory influence of NNMT expression on this transcriptional program.

### 2.6 Targeting NNMT or Prrx1‐SE Interaction Attenuates MMT‐Mediated Scar Progression

**Figure 6:**
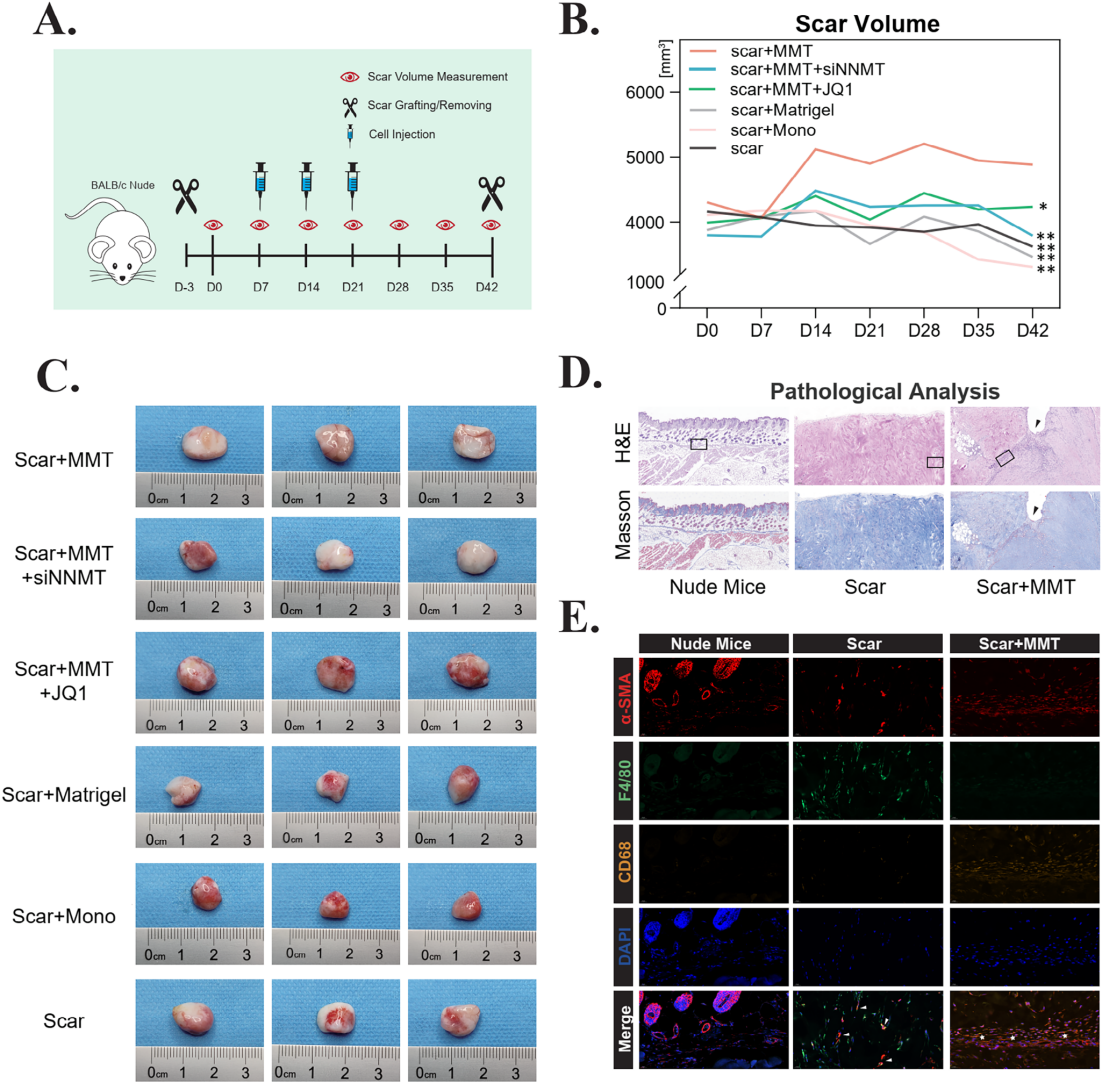
MMT Promotes Scar Progression in a Scar Xenograft Model. A) Schematic of the in vivo experimental design. Hypertrophic scar (HS) tissues from patients were grafted into BALB/c nude mice three days before first volume measurement. MMT cells (or control groups) mixed with Matrigel were injected into the graft on days 7, 14, and 21 (yellow arrows). Scar volume was measured weekly (grey arrows). Mice were sacrificed on day 42 for endpoint analysis. B) Quantification of scar volume changes across treatment groups. Data represent mean ± SD; statistical significance was assessed by one‐way ANOVA (*p < 0.05; *p < 0.01; n = 5). C) Representative macroscopic images of scar tissues from each experimental group. n = 3. D) Representative images of H&E, Masson's trichrome staining of Mude mice skin, engrafted scar, and scar with MMT injection tissues. Black arrows represent injection point. Black black boxes denote the regions magnified in figure (E). Scale bar = 200 µm. n = 5. E) Immunofluorescence staining of α‐SMA (red), F4/80 (green) and CD68 (orange) in Mude mice skin, engrafted scar, and scar with MMT injection tissues. Injected MMT cells are indicated by co‐localization of α‐SMA and CD68 (pentagrams). Murine derived MMT cells are indicated by co‐localization of α‐SMA and F4/80 (arrow). Scale bar = 20 µm. n = 5. Abbreviations: MMT, macrophage‐myofibroblast transition; Mono, monocyte.

To validate our findings in vivo, we established a scar xenograft model using BALB/c nude mice. As illustrated in the schematic diagram, scar tissues were grafted onto mouse flanks, followed by three sequential injections of experimental cells into the graft site (**Figure** **6**A). Scar volume measurements revealed distinct growth patterns: both cell‐injected groups and Matrigel‐only control groups showed initial volume increases peaking at day 14, likely due to the filling effect of cellular material and Matrigel matrix. However, while control groups stabilized thereafter, MMT cell‐injected scars exhibited sustained progressive growth. Importantly, this scar‐promoting effect was abolished by either NNMT inhibition in MMT cells or JQ1‐mediated disruption of Prrx1‐SE interactions (Figure 6B,C).

Pathological analysis confirmed persistent survival of injected MMT cells, validating their contribution to fibrotic progression (Figure 6D,E, pentagram). Notably, IF staining further revealed infiltrating murine macrophages undergoing MMT, which differentiated into myofibroblasts within engrafted scar tissue, demonstrating endogenous MMT occurrence in vivo (Figure 6D,E, triangle).

**Figure 7:**
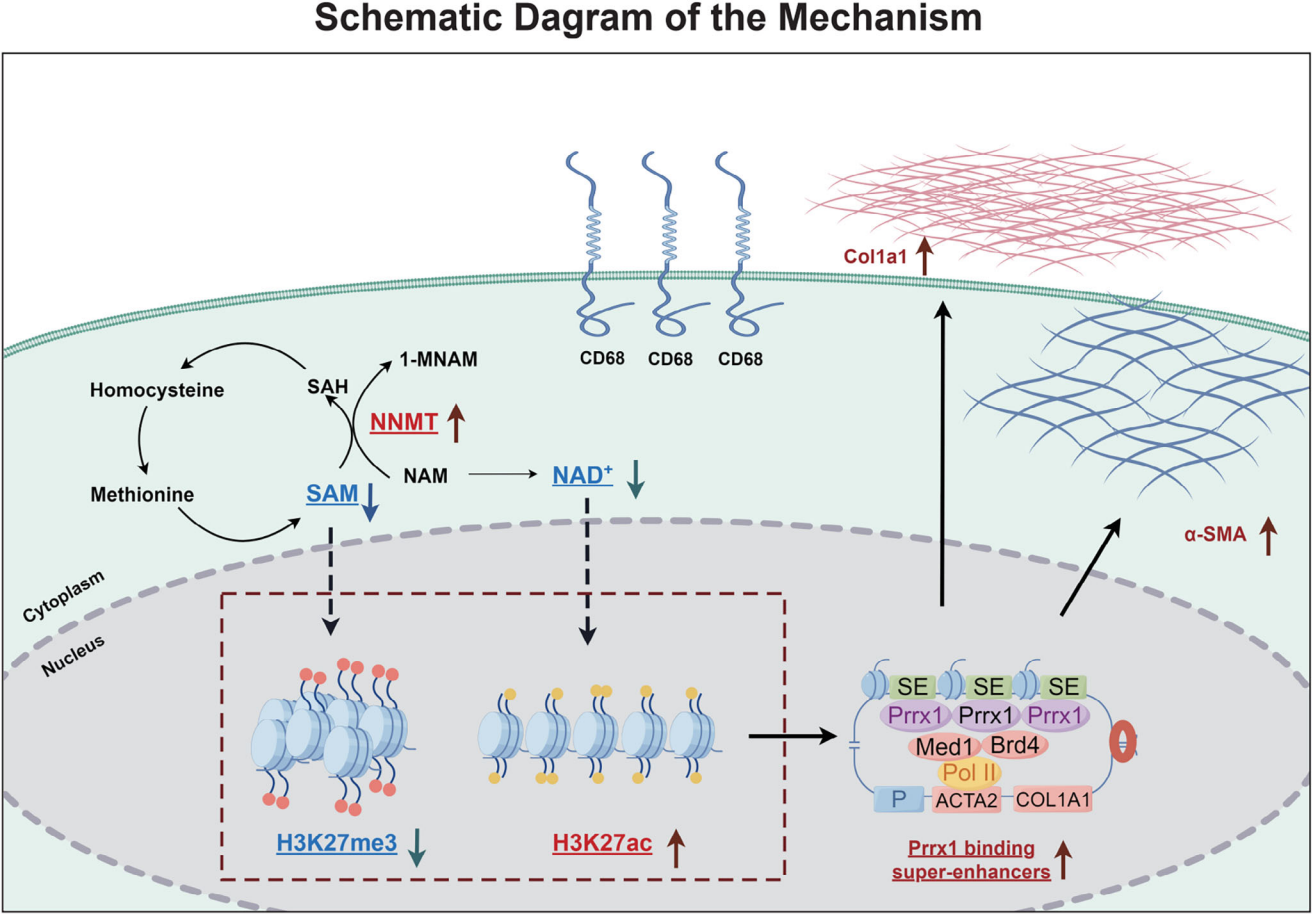
Proposed Mechanism of nicotinamide N‐methyltransferase (NNMT)‐Driven Metabolic‐Epigenetic Crosstalk in Macrophage‐Myofibroblast Transition (MMT). Schematic model depicting the molecular cascade: Upregulated NNMT depletes *S*‐adenosylmethionine (SAM) and nicotinamide adenine dinucleotide (NAD^+^), leading to epigenetic reprogramming via H3K27ac accumulation at the promoter of the master transcription factor paired related homeobox 1 (Prrx1). This chromatin remodeling drives Prrx1 overexpression, enabling its binding to super‐enhancers (SEs) to activate a pro‐fibrotic transcriptional program that promotes myofibroblast lineage commitment and hypertrophic scar progression. Abbreviations: NNMT, nicotinamide N‐methyltransferase; SAM, *S*‐adenosylmethionine; NAD^+^, nicotinamide adenine dinucleotide; H3K27ac, histone H3 lysine 27 acetylation; SE, super‐enhancer.

Based on these findings, we propose a mechanistic model in which MMT progression is driven by NNMT‐mediated metabolic and epigenetic reprogramming through three key steps: 1) NNMT upregulation depletes SAM and NAD^+^ pools; 2) the resulting metabolic changes enhance Prrx1 expression via H3K27ac‐mediated epigenetic activation at its promoter region; 3) the overexpressed Prrx1 binds to SEs to drive myofibroblast lineage specification and scar progression (**Figure** **7**).

## 3 Discussion

The traditional paradigm posited myofibroblasts as terminally differentiated cells derived exclusively from resident fibroblasts. However, this concept has been transformed by recent advances in single‐cell biology, which have revealed myofibroblast differentiation to be a highly plastic and reversible process that can be initiated in diverse progenitor cell populations, including pericytes, endothelial cells, and mesenchymal stromal cells, through distinct molecular pathways.^[^ ^17^, ^18^ ^]^ Our study, in conjunction with emerging evidence, has identified myeloid cells as a previously underappreciated source of myofibroblasts through a specialized phenotypic transition mechanism.^[^ ^5^, ^6^, ^7^, ^9^, ^19^ ^]^ While circulating fibrocytes have been shown to acquire fibrotic markers (α‐SMA and Col1a1) in pathological conditions, suggesting potential functional similarities with MMT cells,^[^ ^20^ ^]^ scRNA‐seq analysis revealed minimal overlap between these populations (Figure S4D–F, Supporting Information). Intriguingly, we observed significant upregulation of pro‐inflammatory markers (*CCL2, CXCL2, and NFKBIA*) in MMT cells compared to conventional fibroblasts and myofibroblasts, mirroring the signature of inflammatory myofibroblast progenitors implicated in scar pathogenesis (Figure S6F, Supporting Information).^[^ ^21^ ^]^ These findings suggest a potential link between MMT cells and inflammatory myofibroblast progenitors, warranting further investigation into their functional relationship and relative contributions to fibrotic and inflammatory processes in fibrotic diseases.^[^ ^22^ ^]^

Histone modifications have emerged as critical epigenetic regulators of myofibroblast activation and fibrotic progression.^[^ ^9^ ^]^ Among various histone marks, H3K27ac has been consistently associated with pro‐fibrotic gene activation. For instance, Wang et al. demonstrated that H3K27ac enrichment at the promoters of *ACTA2* and *PCNA* drives fibroblast activation and proliferation in renal fibrosis.^[^ ^23^ ^]^ Similarly, H3K27ac‐mediated epigenetic regulation at the *PLK1* promoter has been shown to facilitate hepatic stellate cell transdifferentiation into myofibroblasts.^[^ ^24^ ^]^ Conversely, H3K27me3 generally functions as a transcriptional repressor with anti‐fibrotic properties.^[^ ^25^, ^26^ ^]^ Our findings reveal that NNMT upregulation orchestrates a pro‐fibrotic epigenetic landscape by simultaneously promoting H3K27ac and suppressing H3K27me3 modifications.

Our results and previous researches have shown that NNMT can influence various histone modifications (including H3K27, H3K4, H3K9, etc.).^[^ ^27^ ^]^ Mark et al. pointed out that “NNMT does not regulate all histone methylation events or global DNA methylation, which suggests that the enzyme selectively impacts some, but not all cellular methylation pathways, possibly depending on the relative Km and Ki values of individual methyltransferase enzymes (including NNMT itself) for SAM and SAH, respectively”.^[^ ^27^ ^]^ Therefore, further systematic investigation to fully elucidate the preference and complexity of epigenetic network is required in future research. Furthermore, although systemic administration of an NNMT inhibitor has demonstrated safety in obese mouse model, the efficacy and pharmacokinetic profile of localized NNMT inhibition in HS remain unvalidated and warrant dedicated preclinical assessment.

SEs represent a specialized class of cis‐regulatory elements that establish positive feedback loops to promote robust gene expression. This process is mediated by the formation of phase‐separated condensates comprising mTFs, mediator complexes, and specific histone modifications, particularly H3K27ac.^[^ ^16^, ^28^, ^29^ ^]^ The dynamic interplay between SEs and mTFs orchestrates cell fate determination by regulating lineage‐specific gene expression programs.^[^ ^30^ ^]^ For instance, in T cell development, RORγt and FOXP3 engage with SEs to initiate Th17 and Treg cell differentiation, respectively. Analogously, Prrx1, as a myofibroblast‐specific mTF, promotes the expression of α‐SMA and ECM components through SE binding.^[^ ^15^ ^]^ Previous investigations have demonstrated that Prrx1 knockout impairs wound healing, characterized by diminished myofibroblast populations, reduced α‐SMA expression, and attenuated epidermal and dermal thickening. Conversely, Prrx1 overexpression accelerates wound closure but predisposes to pathological scarring through persistent myofibroblast activation.^[^ ^15^, ^31^ ^]^ Our findings align with these observations, showing that pharmacological disruption of Prrx1‐SE interactions or Prrx1 knockdown effectively attenuates MMT progression and α‐SMA expression. However, the comprehensive SE landscape during MMT remains to be fully elucidated. Furthermore, ChIP‐qPCR analysis revealed significant H3K27ac enrichment at both the PRRX1 promoter and associated SE regions during MMT (Figure 4K,L), raising intriguing questions about potential PRRX1 transcriptional addiction mechanisms in MMT progression that warrant further investigation.

Emerging evidence has established the presence of MMT cells in various fibrotic pathologies, particularly renal fibrosis. Lan et al. demonstrated that MMT cells can constitute up to 69% of the total myofibroblast population in renal fibrosis models and clinical specimens from chronically rejected kidney transplants, with MMT prevalence strongly correlating with renal function deterioration and fibrosis severity.^[^ ^5^, ^32^ ^]^ Furthermore, MMT has been implicated in cancer‐associated fibroblast (CAF) generation in non‐small‐cell lung carcinoma.^[^ ^7^ ^]^ Previous mechanistic studies have primarily focused on Smad3, a key transcriptional cofactor in TGF‐β signaling, which undergoes phosphorylation and nuclear translocation upon TGF‐β receptor activation.^[^ ^5^, ^6^, ^32^, ^33^ ^]^ Downstream effectors of Smad3 in MMT include transcription factors Pou4f1 and Runx1, along with tyrosine protein kinase Src. ^[^ ^5^, ^19^, ^33^ ^]^ Interestingly, while our transcription factor network analysis did not recognize Smad3 as a top‐ranked regulator in MMT cells (Figure 4C), we identified conserved Smad binding elements (SBE, GTCTAGAC) within the SE region of PRRX1 (Figure S6G,H, Supporting Information). This finding aligns with Lee et al.’s report of a functional Prrx1‐Smad3 complex in myofibroblast lineage specification.^[^ ^15^ ^]^ These observations suggest a potential synergistic relationship between Prrx1 and Smad3 signaling in MMT progression, warranting further mechanistic investigation.

In conclusion, our study elucidates a novel pathogenic mechanism in HS formation, centered on the metabolic enzyme NNMT. We demonstrate that NNMT drives MMT through a coordinated metabolic‐epigenetic axis: NNMT‐mediated depletion of SAM and NAD^+^ pools elevate H3K27ac levels, thereby promoting the expression and nuclear localization of the mTF Prrx1 and enhancing its interaction with SEs. These findings establish NNMT as a promising therapeutic target for scar modulation. Future investigations should focus on evaluating the safety profile and therapeutic efficacy of NNMT inhibition, while exploring its potential in anti‐fibrotic strategies targeting MMT progression.

## 4 Experimental Section

### Reagents and Antibodies

Chemical reagents, biochemicals, and antibodies used in this study are listed in Tables S3 and S4 (Supporting Information), including detailed information on manufacturers, catalog numbers, and usage.

### Human Subjects and Tissue Collection

This study was conducted in accordance with the ethical principles of the Declaration of Helsinki and received approval from the Institutional Review Board of the Air Force Medical Center (Protocol No. 2024‐026‐S01). Informed consent was obtained from all participants prior to tissue collection.

Fifteen patients were recruited with HS and fifteen patients with MS who were undergoing scar revision surgery to improve functional mobility. Tissue collection was performed as part of the standard surgical procedure, with no additional interventions performed solely for research purposes. For each patient, paired samples were collected, including: 1) Pathological scar tissue from the surgical excision site; 2) Adjacent normal skin tissue (≥2 cm from scar margin) obtained as part of the surgical margin. All tissue samples were immediately processed according to standardized protocols for subsequent experimental analyses.^[^ ^34^ ^]^

### IHC Staining and Quantitative Analysis

IHC staining was performed using the Histostain‐SP Kit according to the manufacturer's protocol. Formalin‐fixed, paraffin‐embedded tissue samples were sectioned at 5 µm thickness using a rotary microtome. Following deparaffinization and rehydration, antigen retrieval was performed using citrate buffer (pH 6.0) at 95 °C for 20 min (min). Endogenous peroxidase activity was blocked with 3% hydrogen peroxide for 10 min at room temperature. Tissue sections were then incubated with primary antibodies against NNMT or Prrx1 (see Table S4, Supporting Information for antibody details) overnight at 4 °C in a humidified chamber. After washing, sections were sequentially incubated with biotinylated goat anti‐mouse IgG secondary antibody and horseradish peroxidase‐conjugated streptavidin, followed by color development using 3,3′‐diaminobenzidine (DAB) as the chromogen. Nuclei were counterstained with Mayer's hematoxylin. Each experimental group comprised seven patients, with all assessments performed in duplicate.

For quantitative analysis, all slides were independently evaluated by two pathologists blinded to the experimental conditions. Positive staining was defined as clear cytoplasmic (for NNMT) or nuclear (for Prrx1) immunoreactivity. Histochemistry scores (H‐scores) were calculated using the following formula: H‐score = Σ (Pi × Ii), where Pi represents the percentage of positive cells (0‐100%) and Ii denotes the staining intensity (1 = negative/trace, 2 = weak, 3 = moderate, 4 = strong). Based on H‐scores, samples were classified into three categories: negative/low (0‐200), intermediate (201‐300), and high expression (301‐400).^[^ ^35^ ^]^ Digital image analysis was performed using Fiji/ImageJ software (NIH, Bethesda, MD) to quantify the percentage of positive staining area in each section.

### Immunofluorescence (IF) Staining and Confocal Microscopy Analysis

Tissue preparation followed the same fixation and sectioning protocol as described for IHC (Section 4.3). Following antigen retrieval and blocking of endogenous peroxidase activity, sections were incubated with primary antibodies against CD68 and α‐SMA (see Table S4, Supporting Information for antibody details) overnight at 4 °C in a humidified chamber. After thorough washing, sections were incubated with species‐specific secondary antibodies: FITC‐conjugated goat anti‐rabbit IgG and Cy3‐conjugated goat anti‐mouse IgG (Table S4, Supporting Information) for 40 mins at room temperature, protected from light. Nuclei were counterstained with DAPI (4′,6‐diamidino‐2‐phenylindole) for 5 mins. Slides were mounted using ProLong Diamond Antifade Mountant and stored at 4 °C in the dark until imaging. Each experimental group comprised seven patients, with all assessments performed in duplicate.

Immunological evaluation of xenografted scar tissues followed established protocols with specific modifications: primary antibody against F4/80 was incorporated for murine macrophage identification, while secondary antibody applications were adjusted to utilize FITC‐conjugated goat anti‐rat IgG, Cy5‐conjugated goat anti‐mouse IgG, and Cy3‐conjugated goat anti‐rabbit IgG to accommodate multispectral detection requirements.

Fluorescence imaging was performed using a Zeiss LSM880 confocal laser scanning microscope equipped with Airyscan super‐resolution capability. Image acquisition parameters were standardized across all samples, including laser power, gain, and pinhole size. Image analysis and colocalization studies were performed using ZEN Blue software (Carl Zeiss, Germany) and Fiji/ImageJ (NIH, Bethesda, MD).

### mIHC Staining

Tissue sections were processed using a standardized multiplex immunohistochemistry protocol. Briefly, paraffin‐embedded sections were deparaffinized in xylene and rehydrated through a graded ethanol series. Antigen retrieval was performed by microwave heating in citrate buffer (10 mmol L^−1^, pH 6.0) for 20 min. Endogenous peroxidase activity was blocked with 3% hydrogen peroxide in PBS for 25 min at room temperature in the dark. Non‐specific binding sites were blocked with 10% normal goat serum in PBS for 30 min at room temperature. The staining protocol involved sequential rounds of antibody labeling using tyramide signal amplification (TSA): 1) Primary antibody incubation: Anti‐CD68 (1:200) overnight at 4 °C in a humidified chamber; 2) Secondary antibody: HRP‐conjugated goat anti‐rabbit IgG (1:200) for 50 mins at room temperature; 3) TSA development: iF440‐TSA for 10 mins in the dark; (4) Antibody stripping: Microwave heating in citrate buffer (10 mmol L^−1^, pH 6.0) for 10 min. This cycle was repeated for subsequent markers (α‐SMA, NNMT, and Prrx1) using corresponding TSA fluorophores (iF488, iF546, and iF594). Nuclei were counterstained with DAPI (1 µg mL^−1^) for 10 mins at room temperature. Slides were mounted with ProLong Diamond Antifade Mountant and stored at 4 °C until imaging.

Whole‐slide imaging was performed using a high‐resolution digital slide scanner (Pannoramic 250 Flash III, 3DHistech) with a 20× objective lens. Multispectral images were acquired and analyzed using CaseViewer software (3DHistech) and Fiji/ImageJ (NIH, Bethesda, MD). Detection was carried out in duplicate.

### Cell Culture, MMT Induction, and Gene Manipulation

PBMCs were isolated from healthy donor blood samples using density gradient centrifugation with Ficoll‐Paque as previously described.^[^ ^35^ ^]^ Monocytes were subsequently purified from PBMCs using the Pan Monocyte Isolation Kit according to the manufacturer's protocol. Cell viability was assessed using trypan blue exclusion, with >95% viability required for subsequent experiments.

Both primary human monocytes and THP‐1 cells were maintained in RPMI 1640 medium supplemented with 10% heat‐inactivated fetal bovine serum (FBS) and 1% penicillin‐streptomycin at 37 °C in a humidified 5% CO2 atmosphere. To induce MMT, primary monocytes were differentiated by macrophage colony stimulating factor (M‐CSF, 50 ng mL^−1^), transforming growth factor beta (TGF‐β, 80 ng mL^−1^) and platelet‐derived growth factor‐BB (PDGF‐BB, 50 ng mL^−1^) for 6 days (Figure 3A). For THP‐1 cells, Phorbol 12‐myristate 13‐acetate (PMA, 25 nmol L^−1^) and 50 ng mL^−1^ TGF‐β were added to culture media for 3 days, together with TGF‐β (50 ng mL^−1^) treatment for additional 2 days.

NNMT‐specific siRNAs were designed and validated, with target sequences provided in Table S5 (Supporting Information). Cells were seeded in 6‐well plates at 2 × 10^5 cells per well and allowed to differentiate for 24 h. Transfection was performed using Advanced Transfection Reagent with 20 nmol L^−1^siRNA for 72 h. Knockdown efficiency was verified by western blot analysis. Each experimental group contained three biological replicates, with all measurements performed in triplicate.

### ICC and Confocal Microscopy

Cells were seeded onto poly‐L‐lysine‐coated confocal dishes at a density of 5 × 10^4 cells per dish and allowed to adhere overnight. Following MMT induction and subsequent treatment, cells were fixed with 4% paraformaldehyde in PBS for 10 mins at room temperature and permeabilized with 0.5% Triton X‐100 for 10 mins. Non‐specific binding sites were blocked with 2% bovine serum albumin (BSA) in PBS for 1 h at room temperature.

Primary antibodies (see Table S4, Supporting Information for details) were applied overnight at 4 °C in a humidified chamber. After three washes with PBS, cells were incubated with species‐specific secondary antibodies conjugated to FITC or Cy3 for 1 h at room temperature, protected from light. Nuclei were counterstained with Hoechst 33342 (1 µg mL^−1^) for 1 h. Following final washes, samples were stored in PBS at 4 °C until imaging.

Confocal imaging was performed using a Zeiss LSM880 microscope equipped with Airyscan super‐resolution capability. Images were acquired using standardized acquisition parameters across all samples. Image analysis was performed using ZEN Blue software (Carl Zeiss) and Fiji/ImageJ (NIH). Each experimental group contained three biological replicates, with all measurements performed in triplicate.

### Flow Cytometry Analysis

Following experimental treatments, cells were harvested and processed for flow cytometric analysis. Cell viability was assessed using Fixable Viability Dye eFluor 780 according to the manufacturer's protocol, enabling the exclusion of non‐viable cells from subsequent analysis. For intracellular staining of CD68 and α‐SMA, cells were fixed and permeabilized using the Foxp3/Transcription Factor Staining Buffer Set. Fc receptors were blocked using Human TruStain FcX on ice for 20 mins. Cells were stained with FITC‐conjugated anti‐human CD68 and CoraLite Plus 647‐conjugated anti‐α‐SMA antibodies for 30 mins at room temperature, protected from light.

After washing with staining buffer, cells were resuspended in PBS containing 1% BSA and analyzed using a BD LSRFortessa flow cytometer. Data acquisition was performed using FACSDiva software, with at least 20000 events collected per sample. Gating strategy and compensation controls were established using single‐stained samples (Figure S7A,B, Supporting Information). Data analysis was performed using FlowJo software. Each experimental group contained three biological replicates, with all measurements performed in triplicate.

### Western Blot Analysis

Following experimental treatments, cells were harvested by centrifugation at 500 × g for 5 mins at 4 °C and washed three times with ice‐cold PBS. Cell pellets were lysed in RIPA buffer supplemented with protease inhibitor cocktail and 1 mmol L^−1^ phenylmethylsulfonyl fluoride (PMSF) on ice for 15 mins. Lysates were clarified by centrifugation at 13000 × g for 20 mins at 4 °C, and supernatants were collected for protein quantification using the BCA Protein Assay Kit.

Protein samples were mixed with 5× Laemmli buffer, denatured at 95 °C for 8 mins, and resolved on 12.5% SDS‐polyacrylamide gels. Proteins were transferred to PVDF membranes using a semi‐dry transfer system. Membranes were blocked with 5% non‐fat dry milk in TBST for 1 h at room temperature.

Immunoblotting was performed using primary antibodies (see Table S4, Supporting Information) overnight at 4 °C, followed by species‐specific HRP‐conjugated secondary antibodies for 1 h at room temperature. Protein bands were visualized using Pierce ECL Western Blotting Substrate on a Tanon 5200 Chemiluminescent Imaging System. Densitometric analysis was performed using ImageJ software (NIH). Each experiment was independently repeated at least three times.

### ChIP and Quantitative PCR

Chromatin immunoprecipitation was performed using the SimpleChIP Plus Enzymatic Chromatin IP Kit according to the manufacturer's protocol. Briefly, 1 × 10^7 cells per condition were cross‐linked with 1% formaldehyde for 10 mins at room temperature, followed by glycine quenching. Cells were lysed, and chromatin was sheared using ultrasonic processor.

Immunoprecipitation was carried out overnight at 4 °C with 5 µg of specific antibodies against H3K27ac or isotype control IgG (see Table S4, Supporting Information for antibody details). Protein‐DNA complexes were captured using ChIP‐grade protein G magnetic beads. After extensive washing, cross‐links were reversed by incubation at 65 °C for 4 h in the presence of 200 mmol L^−1^ NaCl. Samples were treated with RNase A for 30 mins at 37 °C, followed by proteinase K digestion for 2 h at 65 °C.

DNA was purified using phenol‐chloroform extraction and ethanol precipitation. Quantitative real‐time PCR was performed using SYBR Green Master Mix on a QuantStudio 6 Flex Real‐Time PCR System. Primer sequences targeting specific genomic regions are listed in Table S2 (Supporting Information). Measurements were performed in duplicate.

### Targeted Metabolomics Analysis by LC‐MS

To detected total amount of NAD^+^ and SAM, LC‐MS was carried out. After removing culture medium cells were washed for three times with precooled PBS and were quenched with methanol at ‐80 °C for 20 min. Cells then underwent ultrasonication and centrifuged at low temperature (4 °C, 14,000 rpm, 10 min) twice. Collect the supernatant, evaporate to dryness, and reconstitute with 100 µL of methanol. Add 100 µL of the initial mobile phase containing an internal standard (2,3,3,3‐D4‐alanine at 1 µg mL^−1^) [acetonitrile/5 mmol L^−1^ammonium acetate (adjusted to pH = 3.0 with formic acid), 70:30, v/v]. Vortex for 5 mins, transfer to a 1.5 mL EP tube, and centrifuge at low temperature (4 °C, 14,000 rpm, 10 mins) twice. Transfer the supernatant to the sample vial for analysis. Chromatography was carried out as follows. A Waters XBridge BEHAmide column (2.1 × 100 mm, 2.5 µm, Waters, USA) and a Waters XBridge BEH Amide guard column (2.1 × 5 mm, 2.5 µm, Waters, USA) were used. Column temperature: 35 °C; injector temperature: 4 °C; flow rate: 0.3 mL min^−1^. Mobile phase: A phase: 5 mmol L^−1^ammonium acetate (adjusted to pH = 3.0 with formic acid); B phase: acetonitrile. Gradient elution program: 0–7 min: 70‐40% B; 7–9 min: 40% B; 9–10 min: 40–70% B; equilibrate between injections for 3 min. Injection volume: 10 µL. For mass spectrometry, Electrospray ionization (ESI) was used in positive ion mode with multiple reaction monitoring (MRM) mode for data acquisition. Spray voltage: 4.0 kV; desolvation line temperature: 250 °C; heat block temperature: 400 °C; nebulizing gas (nitrogen) flow rate: 3.0 L min^−1^; drying gas (nitrogen) flow rate: 10 L min^−1^; heating gas flow rate: 10 L min^−1^; collision gas (argon) pressure: 270 kPa. The monitored ion pairs and collision energies for the analytes are shown in Table S6 (Supporting Information). Each experimental group contained three biological replicates, with all measurements performed in duplicate.

### Animal Experiment

The current animal study was conducted in accordance with protocols approved by the ethical review board of the Air Force Medical Center (2024‐026‐S01). Animal experiments were carried out according to protocols.^[^ ^36^ ^]^ Shortly, surgical procedures were carried out under inhaled anesthesia using 2–3% isoflurane in 100% oxygen. Scar tissues were surgically excised, trimmed to remove the epidermis and excess subcutaneous fat under sterile conditions, and cut into uniform pieces measuring 1.5 × 1.5 × 1.5 cm^3^. A total of 30 female BALB/c‐nu/nu mice (HFK Bio‐Technology, Beijing, China), aged 4–6 weeks and weighing 20–25 g, were utilized in the study. The mice were randomly divided into six experimental groups: scar group, scar + monocyte group, scar + Matrigel group, scar + MMT group, scar + MMT + siNNMT group, and scar + MMT + JQ1 group. Surgical procedures in mice were performed under inhaled anesthesia with 2–3% Isoflurane in 100% oxygen. Mice were divided into six groups concerning scar group, scar+monocyte group, scar+Matrigel group, scar+MMT group, scar+MMT+siNNMT group, scar+MMT+JQ1 group (n = 5), and were repeated once. Scar tissues were transplanted into the flanks of the mice. Three days after transplantation, first volume measurement was carried out with a vernier caliper (designated as day 0). Scar volumes were measured every seven days successively. On days 7, 14, and 21, monocytes, MMT cells, MMT cells with NNMT interference, and MMT cells treated with JQ1 were mixed with Matrigel and injected into the scar surface. As a control, Matrigel alone was injected at the corresponding time points. Mice were humanely sacrificed on day 42 for final analysis.

### ScRNA‐seq Data Analysis

The scRNA‐seq dataset used for scar analysis is detailed in Table S1 (Supporting Information). Data preprocessing, including trimming, was performed using the Galaxy platform.^[^ ^37^ ^]^ Barcode ranking was conducted with DropletUtils (Galaxy version 1.10.0+galaxy2), where the lower bound was determined based on the data's Knee and Inflection points (Figure S1, Supporting Information). Subsequent data processing was carried out on the ASAP v7.0 web‐based platform (Swiss Institute of Bioinformatics) using the Seurat pipeline, as previously described.^[^ ^38^ ^]^ Outlier cells were identified and filtered based on thresholds for UMI counts, read counts, and the number of expressed genes. Normalization of the count data was performed using the LogNormalize function in Seurat. To identify highly variable genes (HVGs), the Dispersion function in Seurat was applied, selecting the top 2000 most variable features. The data were then scaled and centered using the Scaling function. Dimensionality reduction, clustering, and visualization were conducted using the Seurat pipeline, including principal component analysis (PCA), t‐SNE, and UMAP. For cell clustering, the first 20 principal components were utilized, with a resolution parameter set to 0.8. Cell clusters were annotated by referencing previously published marker genes (Figures S2 and S3, Supporting Information).^[^ ^39^, ^40^ ^]^ Differentially expressed genes (DEGs) were identified using the Wilcoxon Rank‐Sum test, with significance thresholds set at a p‐value < 0.05 and a fold change > 1.5.^[^ ^41^ ^]^ Functional enrichment analysis was performed using WebGestalt for Gene Set Enrichment Analysis (GSEA), and transcription factor predictions were conducted using the ChEA3 database.

### RNA Velocity Analysis

The raw FASTQ files of scRNA‐seq data were processed using the Cell Ranger pipeline (version 7.1.0), which aligned the sequencing reads to the reference genome and generated BAM files containing cell barcode and UMI information. RNA velocity analysis was performed using Velocyto (version 0.17.17) to quantify the dynamics of gene expression. Velocyto processed the BAM files to generate a LOOM file, which contains information on both spliced and unspliced mRNA transcripts. The RNA velocity results were further analyzed using scVelo (version 0.3.3) to visualize velocity vectors and infer cell fate transitions. The RNA velocity vectors were overlaid on t‐SNE plots to illustrate the directionality of cellular transitions and the underlying transcriptional dynamics.

### Statistical Analysis

Statistical analyses were performed using GraphPad Prism 9 (GraphPad Software). Data are presented as mean ± SD. Normality was assessed using Shapiro‐Wilk test, and homogeneity of variance was confirmed via Brown‐Forsythe test. Group comparisons were analyzed by one‐way ANOVA followed by Bonferroni's post‐hoc test (two‐sided, α = 0.05). Sample sizes (n) for each experiment are specified in figure legends or method section. A p‐value < 0.05 was considered statistically significant.

## Conflict of Interest

The authors declare no conflict of interest.

## Supporting information



Supporting Information

## Data Availability

The data that support the findings of this study are available from the corresponding author upon reasonable request.
