# Supplementary material for: NNMT Orchestrates Metabolic‐Epigenetic Reprogramming to Drive Macrophage‐Myofibroblast Transition in Hypertrophic Scarring
Source: Adv Sci (Weinh). 2025 Nov 10;13(11):e02727. doi: 10.1002/advs.202502727 (PMC12931248; doi:10.1002/advs.202502727)
Supplement: Supplementary file 1 — Supporting Information [file ADVS-13-e02727-s001.docx]

**Supporting Information:**


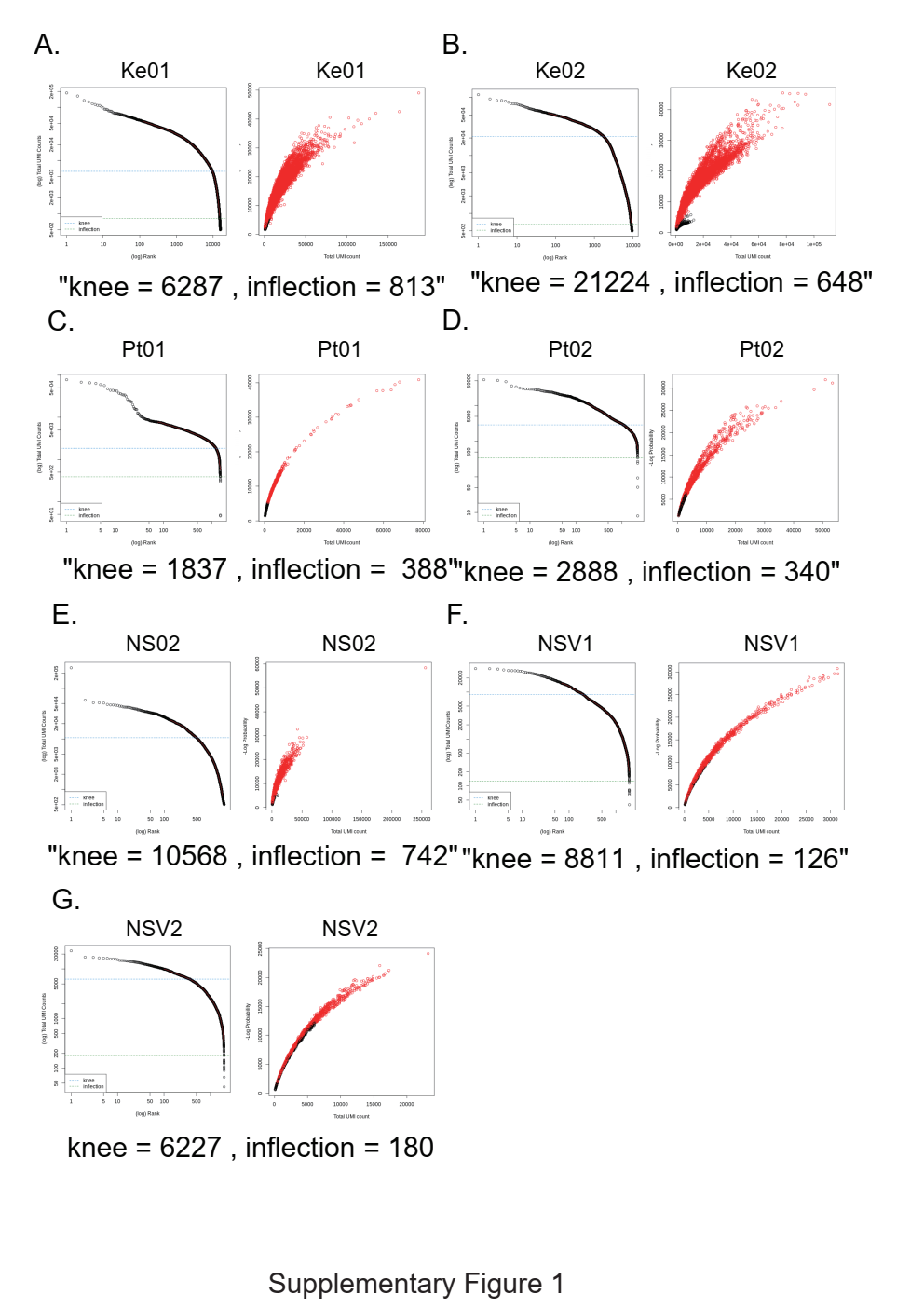


Figure S1:

A-G. Knee and Inflection points of sample Ke01, Ke02, Pt01, Pt02, NS02, NSV1, NSV2, respectively.


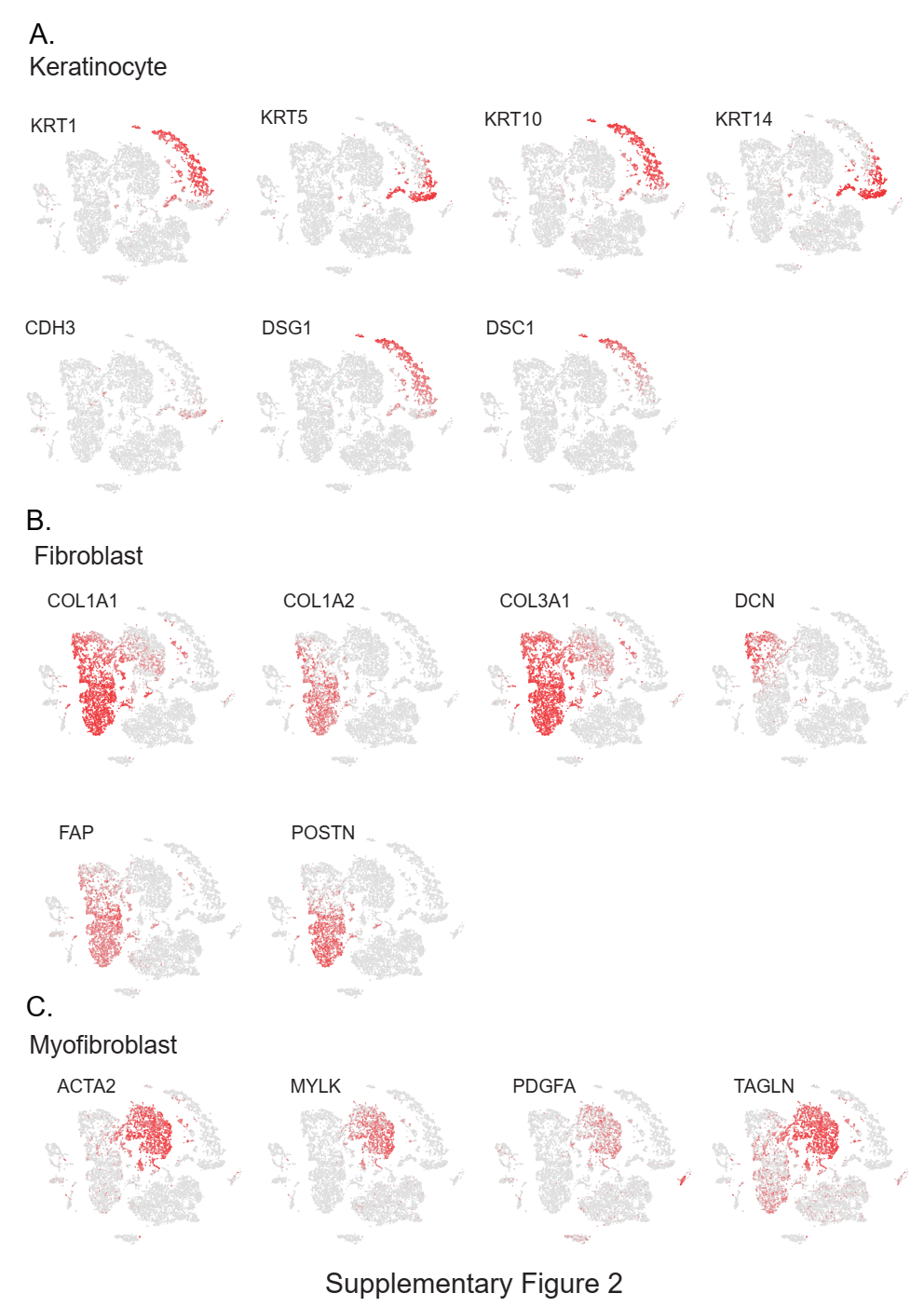


Figure S2:

A-C. Cluster-specific marker genes of keratinocyte, fibroblast, and myofibroblast, respectively.


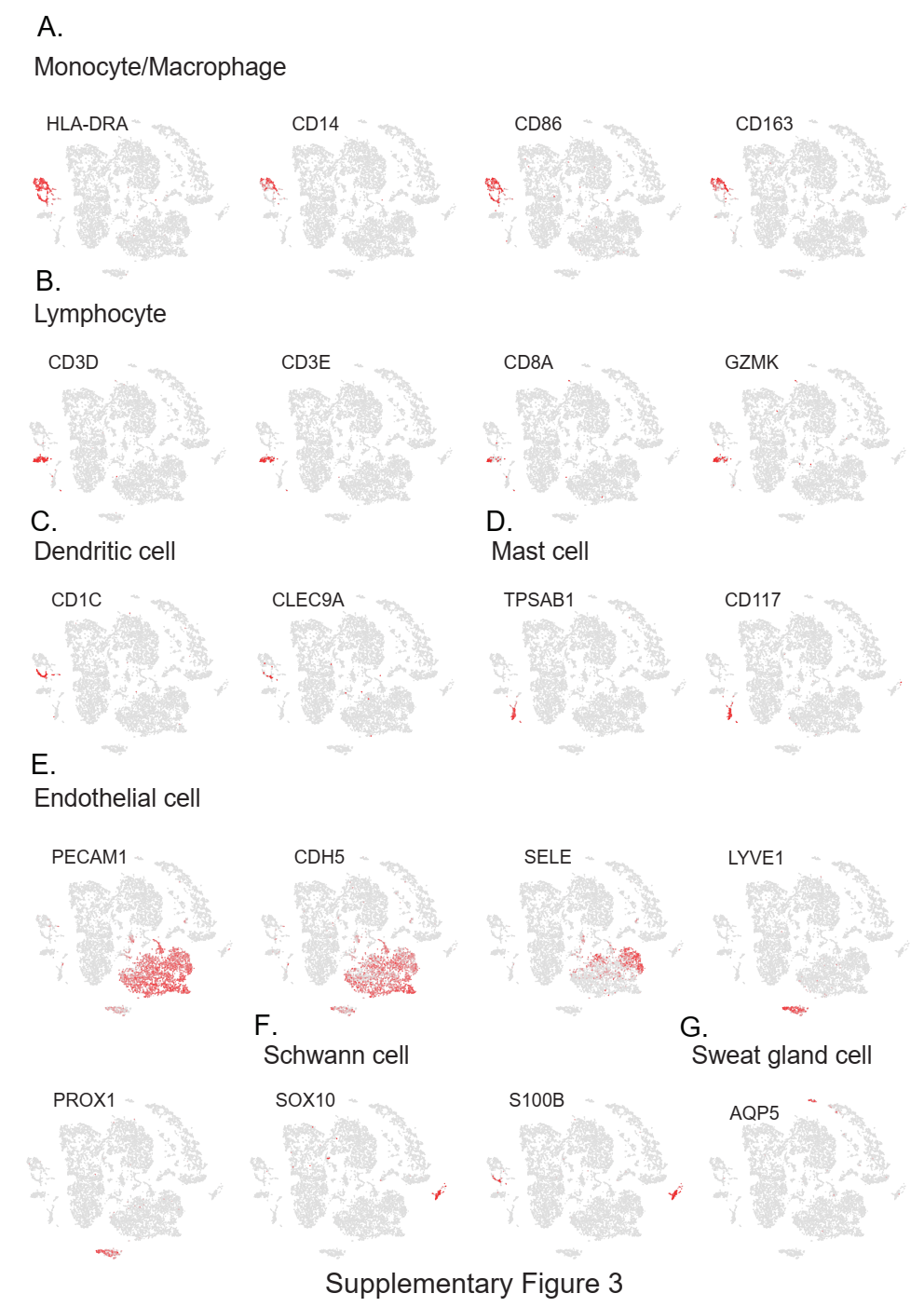


Figure S3:

A-G. Cluster-specific marker genes of monocyte/macrophage, lymphocyte, dendritic cell, endothelial cell, schwann cell and sweat gland cell. respectively.


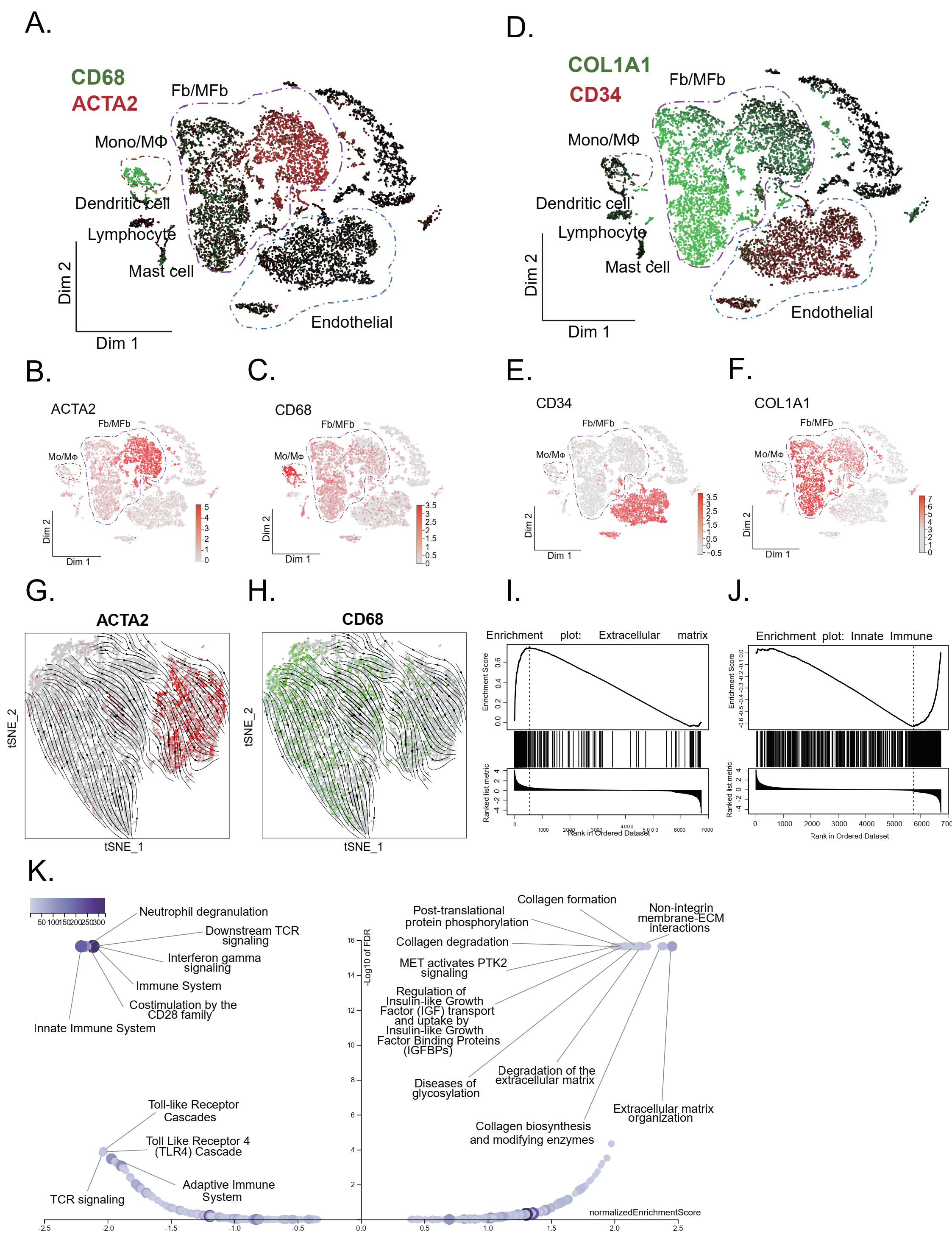


Figure S4:

A-C. CD68 and ACTA2 expression in sc-RNA-seq data. CD68^+^ and ACTA2^+^ cells were mainly distributed in fibroblasts and myofibroblast clusters.

D-F. COL1A1 and CD34 expression in sc-RNA-seq data. COL1A1^+^ and CD34^+^ cells were mainly distributed in monocyte/macrophages and endothelial clusters.

G. RNA velocity vectors overlaid on the t-SNE map, illustrating the trajectory of cells expressing ACTA2.

H.RNA velocity vectors overlaid on the t-SNE map, illustrating the trajectory of cells expressing CD68.

I. Enrichment plot for the R_HSA_1474244, highlighting genes involved in extracellular matrix organization.

J. Enrichment plot for the R_HSA_168249, highlighting genes involved in Innate Immune System.

K. Volcano plot showing the results of Gene Set Enrichment Analysis (GSEA) using the Reactome database.


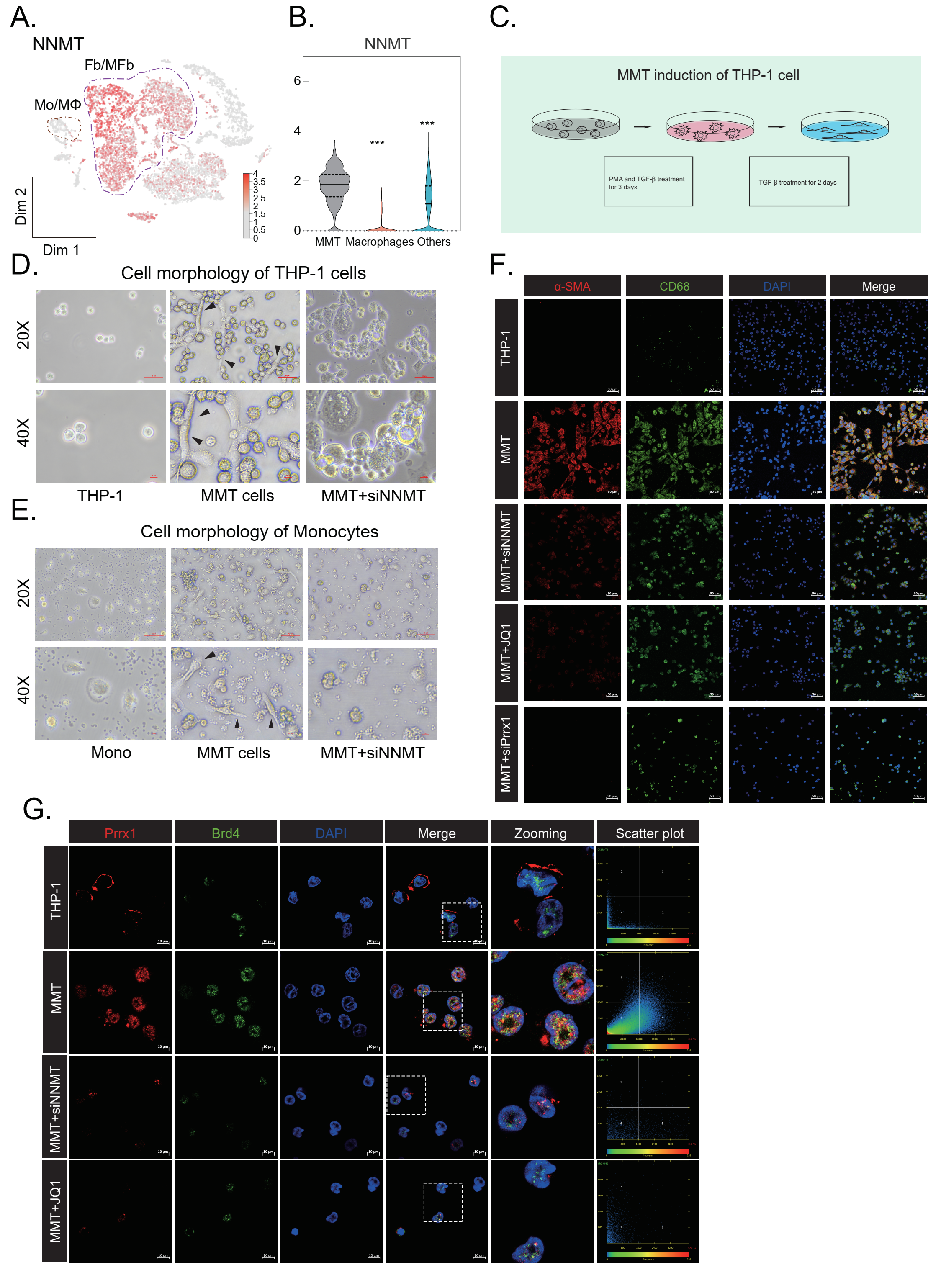


Figure S5:

A. t-distributed Stochastic Neighbor Embedding (t-SNE) visualization of NNMT expression in single-cell RNA sequencing (scRNA-seq) data.

B. Violin plot showing NNMT expression across cell populations: MMT cells, macrophages (MΦ), and other cell types.

C. Schematic of the in vitro induction protocol for differentiating THP-1 cells into MMT cells.

D. Cell morphology after differentiating THP-1 cells into MMT cells. n=3.

E. Cell morphology after differentiating monocytes into MMT cells. n=3.

F. Immunofluorescence images of induced MMT cells (from THP-1 cells), MMT + siNNMT (NNMT knockdown), MMT +JQ1 (BET inhibitor), and MMT + siPrrx1 (Prrx1 knockdown), showing co-expression of α-SMA (red) and CD68 (green). scale bar = 50 μm. n=3.

G. Immunofluorescence imaging showing nuclear co-localization of PRRX1 (red) and BRD4 (green). White boxes indicate enlarged regions, with scatter plots depicting intensity correlations (x-axis: PRRX1; y-axis: BRD4). Cells were treated with siNNMT (NNMT knockdown) or JQ1 (BET inhibitor) during MMT induction. n=3.


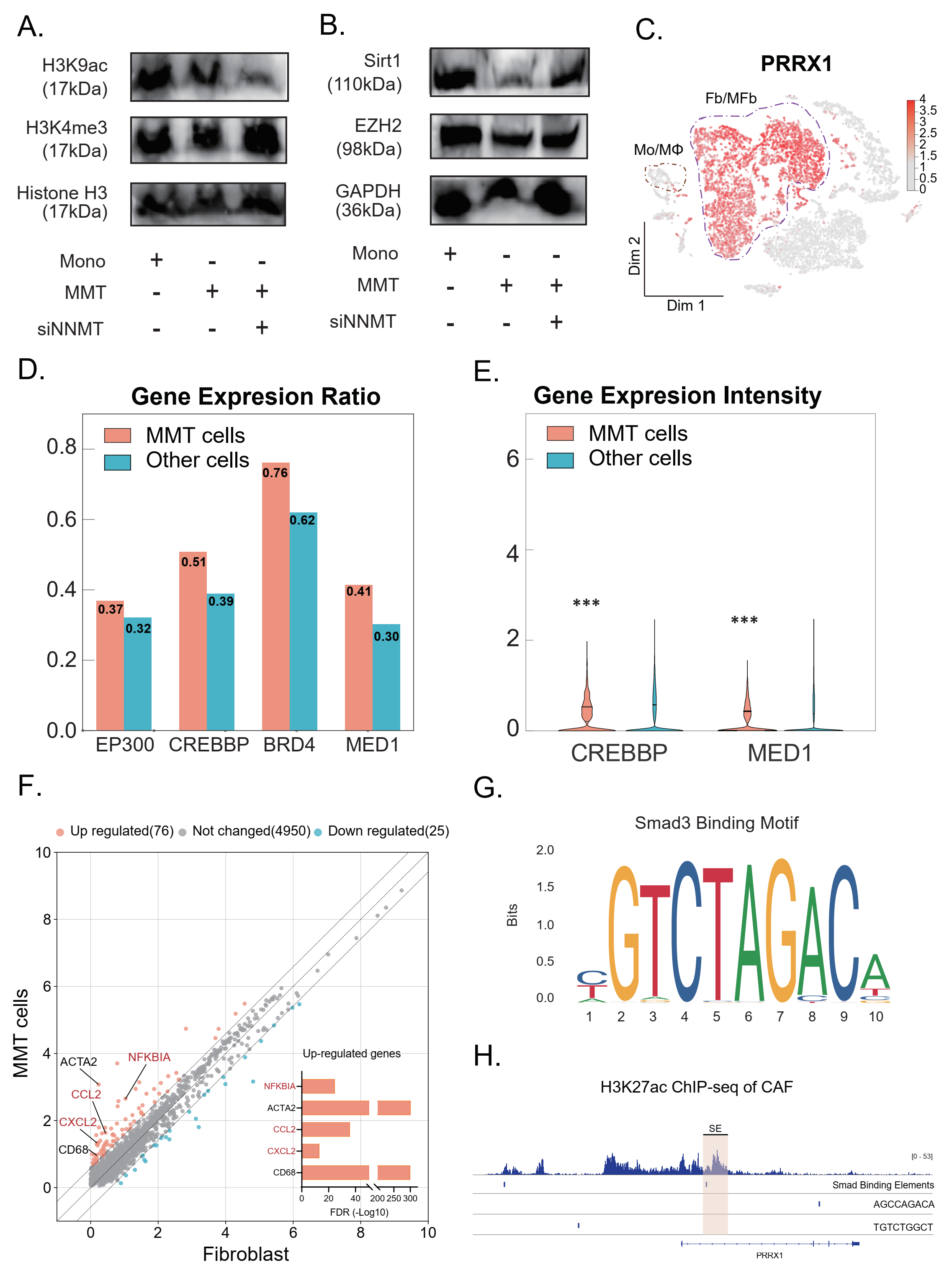


Figure S6:

A. Western blot analysis of H3K9ac, H3K4me3 and Histone H3 in MMT cells. n=3.

B. Western blot analysis of Sirt1, EZH2 and GAPDH in MMT cells. n=3.

C. t-distributed Stochastic Neighbor Embedding (t-SNE) visualization of PRRX1 expression in single-cell RNA sequencing (scRNA-seq) data.

D. Gene expression ratio of EP300, CREBBP, BRD4 and MED1 in MMT cells compared to other cell populations.

E. Gene expression intensity of CREBBP and MED1 in MMT cells compared to other cell populations.

F. Scatter plot comparing gene expression fold changes between MMT cells and firboblast, highlighting CCL2, CXCL2 and NFKBIA (all in red) as a key differentially expressed gene. False discovery rate (FDR) analysis of upregulated genes in MMT cells.

G. Illustration of SMAD3 binding motif.

H. SMAD3 binding motif could be located in SE region of PRRX1 gene in cancer-associated fibroblasts.


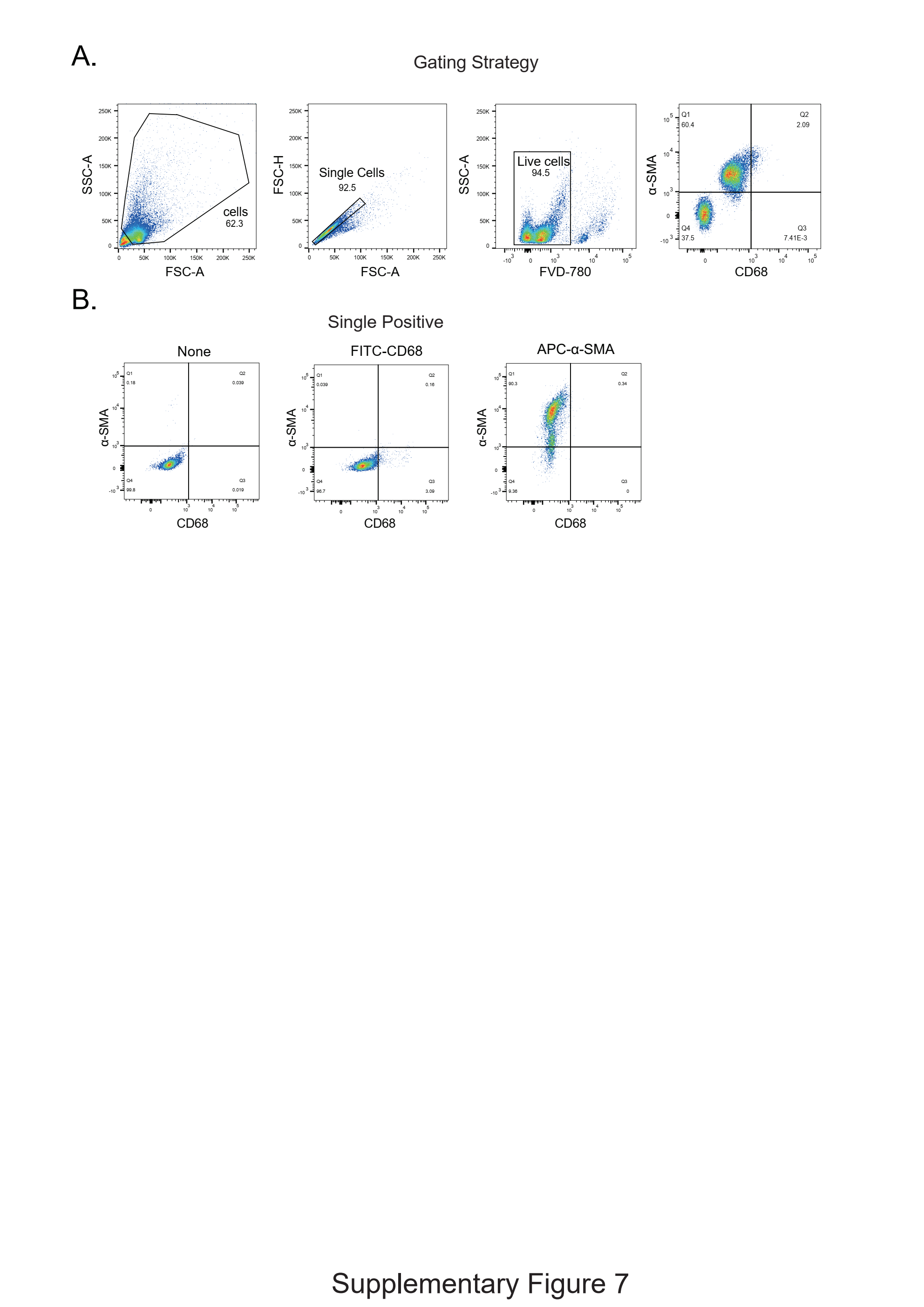


Figure S7:

A. Gating strategy for measuring α-SMA and CD68.

B. Representative image for compensation controls.

Table S1:

**Data sources**

| GEO Number | Related article | Type of analysis |
| --- | --- | --- |
| GSE181297 | Integrated analysis of single-cell and spatial transcriptomics in keloids | scRNA-seq analysis |
| GSM5210583 | Prrx1 is a Master Transcription Factor of myofibroblastic Cancer-Associated Fibroblasts | Chip-seq (CAF) |
| GSM6947034 | Discovery of a druggable copper-signaling pathway that drives inflammation | Chip-seq (Macrophage) |
| GSM3597648 | p63 establishes epithelial enhancers at critical craniofacial development genes | Chip-seq (dermal fibroblast) |

Table S2:

**ChIP-qPCR sequences**

| **Name** | **Sequence** | **Type** |
| --- | --- | --- |
| Prrx1-ChIP | CAGGAGAGGTGATGGCAGAC | Foward |
| Prrx1-ChIP | TGGCTCTCTGAGTTTGCACC | Reverse |

Table S3:

**Reagent Information**

| **Name** | **Company** | **Cat number** | **Usage** |
| --- | --- | --- | --- |
| Human peripheral blood lymphocyte separation medium | Solarbio | P8610-200 | PBMCs Isolation |
| Pan Monocyte Isolation Kit, human | Miltenyi Biotec | 130-096-537 | Monocyte Isolation |
| RPMI 1640 | HyClone | SH30027.01 | Cell culture |
| Penicillin-streptomycin | Solarbio | P1400 | Cell culture |
| Fetal Bovine Serum | Gibico | 12484028 | Cell culture |
| Macrophage colony stimulating factor (M-CSF) | PeproTech | 300-25 | MMT induction |
| Transforming Growth Factor Beta (TGF-β) | PeproTech | 100-21 | MMT induction |
| Platelet-Derived Growth Factor-BB (PDGF-BB) | PeproTech | 100-14B | MMT induction |
| Phorbol 12-myristate 13-acetate (PMA) | MCE | HY-18739 | MMT induction |
| JQ1 | MCE | HY-13030 | Cell treatment: 500nM |
| Ademetionine (SAM) | selleck | S5109 | Cell treatment: 0.1mM |
| Nicotinamide (NAM) | MCE | HY-B0150 | Cell treatment: 1mM |
| SimpleChIP Kit | Cell Signaling Technology | 56383 | ChIP-qPCR: according to user manual |
| Advanced Transfection Reagent | Zeta life | AD600025 | siRNA transfection |
| Hoechst 33,258 | Beyotime | C1011 | ICC:1:1000 |
| Phospophenylmethanesulfonyl fluoride | Servicebio | G2008 | WB:1X |
| BCA Protein Assay Kit | GLPbio | GK10009 | WB: according to user manual |
| Pierce™ ECL Western Blotting Substrate | Thermo Fischer | 32209 | WB: according to user manual |
| Histostain™ - SP Kits | Zhongshan Golden Bridge Biotechnology | SPN-9002 | IHC: according to user manual |
| 2,3,3,3-D4-alanine | Aladdin Scientific | 53795-92-9 | LC-MS |
| S-adenosylmethionine | Aladdin Scientific | R341484 | LC-MS |
| beta-Nicotinamide adenine dinucleotide | Aladdin Scientific | N111610 | LC-MS |
| Fc Receptor Blocking Solution | Biolegend | 422302 | Flow cytometry |
| Foxp3/Transcription Factor Staining Buffer Set | eBioscience | 00-5523-00 | FC: according to user manual |
| Fixable Viability Dye eFluorTM 780 | eBioscience | 65-0865 | FC: according to user manual |
| Apply ProLong™ Diamond Antifade Mountant | Thermo Fisher | P36965 | IF: according to user manual |
| iF440-TSA | Servicebio | G1250 | mIHC:1:500 |
| iF488-TSA | Servicebio | G1231 | mIHC:1:500 |
| iF546-TSA | Servicebio | G1251 | mIHC:1:500 |
| iF594-TSA | Servicebio | G1242 | mIHC:1:500 |
| Matrigel | Corning | 356234 | Animal experiments |
| Isoflurane | RWD Life Science | R510-22-10 | Animal experiments |

Table S4:

:

**Antibody Information**

| **Name** | **Company** | **Cat number** | **Usage** |
| --- | --- | --- | --- |
| CD68 (D4B9C) XP® Rabbit mAb | Cell Signaling Technology | 76437 | IF:1:200  ICC:1:200 |
| Anti-alpha SMA antibody Mouse mAB | Abcam | ab7817 | IF:1:200  ICC:1:200 |
| Anti-Histone H3 (acetyl K27) antibody-ChIP Grade | Abcam | ab4729 | WB:1:1000 |
| TriMethyl-Histone H3 (Lys27) Rabbit mAb | Zen-bio | R26242 | WB:1:1000 |
| TriMethyl-Histone H3 (Lys4) Rabbit pAb | Zen-bio | 502357 | WB:1:1000 |
| TriMethyl-Histone H3 (Lys4) Rabbit pAb | Zen-bio | 340016 | WB:1:1000 |
| Ezh2 (D2C9) XP® Rabbit mAb | Cell Signaling Technology | 5246 | WB:1:1000 |
| [KO] SIRT1 Mouse mAb | Zen-bio | 240048 | WB:1:1000 |
| Histone H3 (1B1B2) Mouse mAb | Cell Signaling Technology | 14269 | WB:1:1000 |
| PRRX1 Mouse Monoclonal Antibody | Origene | TA803116 | WB:1:1000;  IHC:1:200  mIHC:1:200  ICC:1:200 |
| NNMT Mouse Monoclonal Antibody | Origene | TA502624 | WB:1:1000;  IHC:1:200  mIHC:1:200  IF:1:200 |
| BRD4 Rabbit mAb | Zen-bio | R381011 | WB:1:1000;  ICC:1:200 |
| CoraLite® Plus 647-conjugated smooth muscle actin specific Monoclonal antibody | ProteinTech | CL647-67735 | FC: according to user manual |
| FITC Plus Anti-Human CD68 | ProteinTech | FITC-65202 | FC: according to user manual |
| FITC-labeled goat anti-rabbit IgG | Servicebio | GB22303 | IF:1:150 |
| cy3-labeled goat anti-mouse IgG | Servicebio | GB21301 | IF:1:150 |

Table S5:

**siRNA sequences**

| **Name** | **Sequence** | **Type** |
| --- | --- | --- |
| siNNMT-1 | CUAUGUGUGUGAUCUUGAA(dT)(dT) | Sense |
| siNNMT-1 | UUCAAGAUCACACACAUAG(dT)(dT) | Antisense |
| siNNMT-2 | CGCUCAAGAGCAGCUACUA(dT)(dT) | Sense |
| siNNMT-2 | UAGUAGCUGCUCUUGAGCG(dT)(dT) | Antisense |
| siNNMT-3 | GCUCCUCUCUGCUUGUGAA(dT)(dT) | Sense |
| siNNMT-3 | UUCACAAGCAGAGAGGAGC(dT)(dT) | Antisense |

Table S6:

The monitored ion pairs and collision energies for the analytes

| **No** | **Name** | **Abbreviation** | **Precursor ion**  **(m/z)** | **Collision Energy**  **(eV)** | **Product ion**  **(m/z)** |
| --- | --- | --- | --- | --- | --- |
| 1 | S-adenosylmethionine | SAM | 398.9 | -15 | 249.9 |
| 2 | beta-Nicotinamide adenine dinucleotide | NAD | 663.7 | -46 | 135.9 |
| IS | 2,3,3,3-D4-Alanine | D4-Ala | 94.1 | -13 | 48.2 |
